# PIM1 accelerates prostate cancer cell motility by phosphorylating actin capping proteins

**DOI:** 10.1186/s12964-020-00618-6

**Published:** 2020-08-08

**Authors:** Niina M. Santio, Veera Vainio, Tuuli Hoikkala, Kwan Long Mung, Mirka Lång, Riitta Vahakoski, Justyna Zdrojewska, Eleanor T. Coffey, Elena Kremneva, Eeva-Marja Rainio, Päivi J. Koskinen

**Affiliations:** 1 Section of Physiology and Genetics, Department of Biology, University of Turku, Vesilinnantie 5, FI-20500 Turku, Finland; 2 Turku Bioscience, University of Turku and Åbo Akademi University, 20520 Turku, Finland; 3 Institute of Biotechnology, University of Helsinki, 00014 Helsinki, Finland

**Keywords:** PIM kinases, Capping proteins, CAPZ, Actin, Migration, Prostate cancer

## Abstract

**Background:**

The PIM family kinases promote cancer cell survival and motility as well as metastatic growth in various types of cancer. We have previously identified several PIM substrates, which support cancer cell migration and invasiveness. However, none of them are known to regulate cellular movements by directly interacting with the actin cytoskeleton. Here we have studied the phosphorylation-dependent effects of PIM1 on actin capping proteins, which bind as heterodimers to the fast-growing actin filament ends and stabilize them.

**Methods:**

Based on a phosphoproteomics screen for novel PIM substrates, we have used kinase assays and fluorescence-based imaging techniques to validate actin capping proteins as PIM1 substrates and interaction partners. We have analysed the functional consequences of capping protein phosphorylation on cell migration and adhesion by using wound healing and real-time impedance-based assays. We have also investigated phosphorylation-dependent effects on actin polymerization by analysing the protective role of capping protein phosphomutants in actin disassembly assays.

**Results:**

We have identified capping proteins CAPZA1 and CAPZB2 as PIM1 substrates, and shown that phosphorylation of either of them leads to increased adhesion and migration of human prostate cancer cells. Phosphorylation also reduces the ability of the capping proteins to protect polymerized actin from disassembly.

**Conclusions:**

Our data suggest that PIM kinases are able to induce changes in actin dynamics to support cell adhesion and movement. Thus, we have identified a novel mechanism through which PIM kinases enhance motility and metastatic behaviour of cancer cells.

Video abstract

**Graphical abstract:**

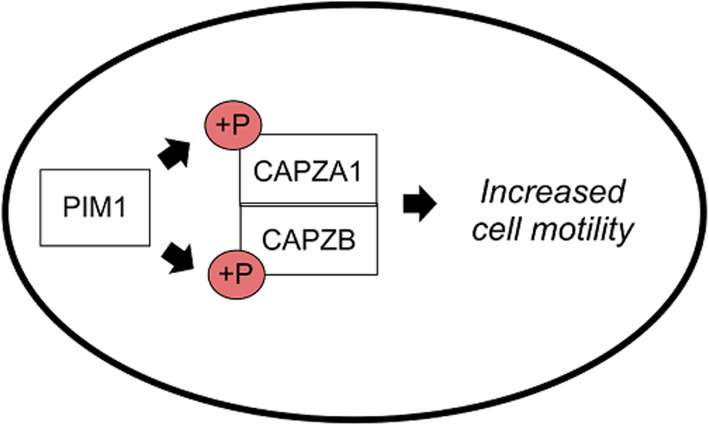

## Background

The hallmarks of malignant cancer cells that distinguish them from healthy cells include increased motility and an enhanced ability to invade other tissues and form metastatic colonies [1]. Cellular movements are normally strictly controlled by a network of intra- and extracellular components, which coordinate cell adhesion, spreading, polarity and locomotion. These include cadherin-like proteins that are responsible for adherent cell-cell junctions, and transmembrane integrins that connect the intracellular molecules to the extracellular matrix [2, 3]. However, the main forces driving cell locomotion are dependent on actin dynamics: the constant polymerization and depolymerization of actin filaments [4]. This leads to changes in cellular protrusions such as filopodia, lamellipodia as well as retraction fibers, which are important mediators of both normal and cancer cell movement [5].

Actin polymerization is regulated by several types of actin-binding proteins, such as the capping proteins (CPs) [5]. They bind as heterodimers to the fast-growing ends of the actin filaments, and thereby prevent both filament assembly and disassembly [6]. CP subunits are evolutionarily conserved, but their number varies between different species [7]. Originally, they were identified in the Z disks of striated muscles, but they have since been found in other muscle and non-muscle tissues as well [7–9]. CP alpha 1 and 2 subunits are expressed in both types of tissues, while alpha 3 is a germ cell-specific subunit [9–11]. Three different splice variants have been identified for the beta subunit, of which beta 1 is localized at the Z disks, and beta 2 at the intercalated disks and cell periphery in the muscles [12]. The third variant, beta 3, is a testis-specific isoform similar to alpha 3 [13]. Despite their importance in skeletal muscle, various functional roles have also been reported in other tissues, such as cardiac tissue, the nervous system and cancer [14–21].

The role of CPs in cancer has remained unclear due to contradictory results with different subunits. While the beta subunit has been reported to act as an oncogene, variable but mainly tumor suppressive roles have been suggested for the alpha subunits [17–21]. These opposite effects are surprising due to the heterodimeric mode of CP action. However, as the previous data are mostly based on silencing or overexpressing of individual subunits, they may not fully reflect the impact of the heterodimers. It is also likely that CP activity is context-dependent, as suggested by the differential expression of their distinct subunits. In addition, CPs are known to be targeted by post-translational modifications, such as phosphorylation by protein kinase Cε and casein kinase 2, both of which reduce actin capping activity [14, 22].

In this study, we have identified the CP alpha 1, alpha 2 and beta 2 subunits as novel substrates for the oncogenic PIM1 kinase. The three serine/threonine-specific PIM family kinases regulate cell growth, metabolism, and motility, and have also neuronal functions [23–26]. They have been implicated in both hematopoietic malignancies and solid cancers [23, 27, 28], and are therefore promising targets for cancer therapy. The effects of PIM kinases on cancer cell motility have been extensively studied in prostate cancer, where they have been shown to increase migration, invasion and adhesion of cultured cells, and enhance tumor angiogenesis and metastasis *in* vivo [29–32]. The pro-migratory effects of PIM kinases have been connected to phosphorylation-dependent activation of several substrates such as NOTCH1, NFATC1 and EIF4B, or inactivation of tumor suppressive factors such as FOXP3 [31, 33–36]. However, the previously identified PIM substrates do not regulate cellular movements by directly interacting with the actin cytoskeleton. Here we have used a dual expression plasmid to simultaneously study the phosphorylation-dependent effects of both CP alpha 1 and beta 2 subunits on prostate cancer cell motility. We demonstrate that their phosphorylation promotes adhesion and migration of cultured cells, and also decreases their ability to protect actin filament ends from disassembly in vitro. Thus, CP phosphorylation is expected to increase actin dynamics and thereby enhance the motility of prostate cancer cells.

## Methods

### Cloning and mutagenesis

To create cDNA libraries, total mRNA was isolated with Tri Reagent® (#T9424, Sigma-Aldrich, St Louis, MI, USA) from mouse tissue or human PC-3 prostate cancer cells, after which cDNA synthesis was performed using the first strand cDNA synthesis kit (#K1612, Thermo Fisher Scientific, Waltham, MA, USA). The cDNAs of interest were subcloned into pGEM-T-Easy vector (Promega, Madison, WI, USA) by using PCR with gene-specific primers. Further subclonings were performed either by PCR or by digestion with restriction enzymes. The gene-specific cloning and sequencing primers, and the detailed design of constructs are shown in Additional file 1: Tables S1 and S2.

For in vitro kinase assays with bacterially produced proteins, mouse *capza1* or human *CAPZA2* cDNAs were inserted together with mouse *capzb2* cDNA into the dual expression vector pRFSDuet-1 (shortened as “Duet”, #71341, Merck Millipore, Burlington, MA, USA), so that alpha subunits were placed into the multiple cloning site (MCS) 1 and the beta subunit into MCS2. The *capzb2* cDNA was also subcloned into pGEX-6P-3 (GE Healthcare Life Sciences, Little Chalfont, UK).

For expression in mammalian cells, His-tagged *capza1* and *CAPZA2* constructs were prepared by subcloning the cDNAs from Duet to the MCS1 of PSF-CMV-CMV-SBFI-UB-PURO - DUAL CMV plasmid (shortened as “Dual-CMV” or “Dual”; #OGS597, Sigma-Aldrich, St. Louis, MI, USA). The *capzb2* cDNA was Flag-tagged by transferring it from pGEX-6P-3 to pFlag-CMV™-2 (#E7033, Sigma-Aldrich), after which it was further subcloned to Dual-CMV MCS2. For creation of GFP-tagged constructs, *capzb2* was transferred from pGEX-6P-3 to pEGFP-C1 (Clontech laboratories Inc., Takara Bio USA, Inc., Mountain View, CA, USA). In addition, GFP was subcloned from pEGFP-C1 into Dual-CMV prior to MCS2 to create a GFP-tagged Dual-CMV empty vector or a vector expressing Capza1 and GFP-tagged Capzb2.

Site-directed mutagenesis of mouse *capza1* and *capzb* genes was performed by Ultra Pfu DNA polymerase (Stratagene, San Diego, CA, USA) according to Manufacturer’s protocol. The primers used are described in Additional file 1: Table S3.

The short isoform of the murine Pim1 protein cap away was expressed in bacterial cells from the pGEX-2T-Pim1 vector as previously described [34]. The human PIM1 protein was expressed in bacterial cells from the pGEX-6P-1-PIM1 vector or in mammalian cells from the pcDNA™3.1/PIM1-V5-His (from here on “pcDNA-PIM1”) construct or Tag-PIM1-RFP (from here on “PIM1-RFP”), which along with the GST-tagged murine Notch1 intracellular domain (ICD) and the control plasmids have been described previously [33].

### Protein production

For protein production, all bacterial constructs were expressed in the BL21 *E. coli* strain. Production of GST-tagged murine Pim1 or human PIM1 has been described previously [34, 37]. For production of heterodimers of Capza1 or CAPZA2 with Capzb2, cell pellets were suspended in ice-cold lysis buffer (50 mM Tris-HCl pH 7.5, 250 mM NaCl, 10 mM imidazole). Protease activity was inhibited by aprotinin (1 μg/ml) or PMSF (5 μM). Protein purification was performed by rotating the lysate for 30 min at + 4 °C with 50% HisLink™ resin (Promega). Thereafter samples were washed four times in washing buffer (10 mM Tris-HCl, pH 7.5, 20 mM imidazole) and rotated for 30 min at + 4 °C with elution buffer (10 mM Tris-HCl, pH 7.5, 300 mM Imidazole, 250 mM NaCl). The His-tagged alpha subunits formed heterodimers with the beta subunits, so both subunits could be simultaneously isolated. Protein samples were separated by SDS-PAGE and visualised by Page Blue™ Protein Staining Solution (Thermo Fisher Scientific).

### PIM1 substrate screening, in vitro kinase assays and mass spectrometry

Potential Pim1 substrates were identified using an in vitro screen as previously described [38]. Briefly, rat cortex was homogenized in kinase buffer and phosphorylated in vitro at 30 °C for 45 min using recombinant murine Pim1 kinase in the presence of [γ-^32^P] ATP (PerkinElmer Finland Oy, Turku, Finland). Protein extract was loaded onto a dry polyacrylamide gel strip with an immobilized pH gradient of 4–7, according to the manufacturer’s instructions (Amersham Biosciences, Uppsala, Sweden). Proteins were separated in the first dimension by isoelectric focusing overnight at 3500 V, followed by two-dimensional separation on 12% SDS-PAGE, silver staining and autoradiography. Reduction, alkylation, and in-gel trypsin digestion of the proteins were performed as described previously [39]. Peptides were extracted into 5% HCOOH, 50% CAN, concentrated using SpeedVac vacuum and desalted with ZipTips with C18 resin (Millipore). After digestion, peptides were mixed 1:1 with matrix α-ciane-4-hydroxycinnamic acid (HCCA) dissolved in 70% ACN/0.1% TFA and spotted onto a stainless steel target plate. Mass spectrometry (MS) analysis was undertaken with a MALDI-TOF/TOF Ultraflex II mass spectrometer (Bruker Daltonics, Billerica, MA, USA). Spectra were internally calibrated with peptides from trypsin autolysis (M + H^+^ = 842.509, M + H^+^ = 2211.104). The most abundant peptide ions were then subjected to fragmentation analysis (MS/MS) to provide information for use in determining the peptide sequence. Data were processed by Analyst QS software (Applied Biosystems, Foster City, CA, USA) and matched to the SwissProt protein database using the MASCOT algorithm.

Phosphorylation of putative substrates by human PIM1 was validated by radioactive in vitro kinase assays, as described previously [37]. Shortly, 0.5–2.0 μg of PIM1 and its substrate were used in each reaction. Samples were separated by SDS-PAGE and stained by Page Blue™ protein staining solution (#24620, Thermo Fisher Scientific). Results were analysed by autoradiography and quantitated by the ChemiDoc™ MP Imaging System with Image Lab software Version 4.0 (Bio-Rad Laboratories, Inc., Hercules, CA, USA) and ImageJ/Fiji software (1.48 s, Fiji, Wayne Rashband, National Institutes of Health, Bethesda, MD, USA).

For mass spectrometry of phosphorylated substrates, in vitro kinase assays were prepared similarly to the radioactive assays, but without radiolabelled ATP. After SDS-PAGE, the ProQ® Diamond Phosphoprotein Gel Stain (Thermo Fisher Scientific) was used according to manufacturer’s protocol. In-gel digestion of proteins with trypsin, liquid chromatography-electrospray ionization-tandem mass spectrometry (LC-ESI-MS/MS) with phosphopeptide enrichment and analysis of data have been described previously [39–41]. Similar analyses were performed also with immunoprecipitated cellular proteins.

### In silico analyses for mRNA and proteins

Phosphorylation sites were searched for from the in vitro kinase samples by the PhosphoMotif Finder in the Human Protein Reference Database (http://www.hprd.org/PhosphoMotif_finder/). The in vivo phosphorylation sites were searched for from the PhosphoSitePlus® database (phosphosite.org, Cell Signaling Technology, Inc., Danvers, MA, USA). Gene expression data were obtained from the IST Online™ (ist.medisapiens.com [42];) or betastasis database (betastasis.com; gene expression barblot) [43]. Correlation analyses were performed on gene expression data from the betastasis dataset [43]. Homology comparisons were performed by the Basic Local Alignment Search Tool (National Institutes of Health, Bethesda, MD, USA) using protein sequences from the National Center for Biotechnology Information (National Institutes of Health, Bethesda, MD, USA) and UniProt Knowledgebase [44].

### Cell culture and transfections

The human prostate epithelial adenocarcinoma cell line PC-3 and the carcinoma cell lines DU-145 and LNCAP (from American Type Culture Collection) were maintained in RPMI-1640 medium supplemented with 10% fetal bovine serum, L-glutamine and antibiotics. For transient transfections, cells were plated 24 or 48 h earlier, cultured until ~ 80% confluence and then transfected with the FuGENE® HD Transfection Reagent (Promega) 1:3 to DNA. Approximately 0.5 μg of DNA was used to transfect 100,000 cells. The CP subunits were expressed either by cotransfecting His-tagged Capza1 (Dual-CMV) together with Flag-tagged Capzb2 (pFlag-CMV-2), or by transfecting the Dual-CMV vector encoding both His-tagged Capza1 or Capza2 and Flag-tagged Capzb2. The Capzb2 subunit alone was expressed by the Flag-tagged plasmid pFlag-CMV-2. To inhibit the catalytic activity of PIM kinases, cells were treated with the PIM-selective small molecule inhibitors DHPCC-9 [29, 45], AZD-1208 (Astra Zeneca, Cambridge, UK) or SGI-1776 (#526528, Sigma-Aldrich) at 10 μM concentration in 0.1% DMSO, which alone was used in control samples.

### Nuclear fractionation

Cells cultured on 10 cm plates were lysed in 500 μl of lysis buffer: 10 mM Tris-HCL pH 7.5, 10 mM NaCl, 3 mM MgCl_2_, 0.5% Nonidet P-40, 1 mM PMSF and mini EDTA- free protease inhibitor tablet (Roche, Basel, Switzerland) according to the manufacturer’s protocol. 100 μl of lysate was stored as a whole cell lysate control and heated with 5x Laemmli sample buffer (LSB) for 5 min at + 95 °C. After centrifugation at 500 x g for 5 min at + 4 °C, the supernatant contained the cytoplasm, while the nuclei were in the pellet. The pellets were washed three times with lysis buffer and centrifuged each time at 500 x g for 5 min at + 4 °C, after which they were suspended to 200 μl of lysis buffer. The cytoplasm-containing solution was centrifuged at 12000 x g for 15 min at + 4 °C, after which the supernatant was collected. Nuclear and cytoplasmic lysates were heated for 5 min at + 95 °C with 5x LSB. 30 μl aliquots of samples were loaded to each well on SDS-PAGE and samples were analysed by Western blotting, using nuclear A/C laminin and cytoplasmic beta-tubulin as fraction-specific positive controls (Additional file 1: Table S4).

### Western blotting

Cultured cells were directly lysed into 75–120 μl of hot 2x LSB or lysed for co-immunoprecipitation analyses as described below. In both cases, LSB-containing samples were heated for 5 min at 95 °C. Proteins (20–50 μl/ well) were separated by 10–12% SDS-PAGE. After blotting onto PVDF membrane, samples were stained with primary antibodies (Additional file 1: Table S4) at + 4 °C overnight. Secondary antibody staining (1:1000) was performed for 30 min at RT with HRP-linked goat anti-mouse IgG #7076 or goat anti-rabbit IgG #7074 antibodies (Cell Signaling Technology, Beverly, MA, USA).

### Immunoprecipitations and immunofluorescence

Cellular protein interactions were measured by co-immunoprecipitation (CO-IP) and colocalization analyses, after which they were confirmed by proximity ligation assays (PLA) and fluorescence-lifetime imaging microscopy (FLIM). For IP and CO-IP, PC-3 cells were cotransfected with pcDNA-PIM1, His-tagged Capza1 and Flag-tagged Capzb2. After 48 h, cells were scraped into sterile PBS, centrifuged 300 x g for 3–4 min and resuspended into 5x volume of lysis buffer: 50 mM Tris pH 7.5, 10% glycerol, 100 mM NaCl, 1 mM EDTA, 1% NP-40, 50 mM NaF, 250 μM β-glycerophosphate, 1 mM Na_3_VO_4_ and mini EDTA-free protease inhibitor tablet (Roche) according to the manufacturer’s protocol. Samples were incubated on ice for 60 min, vortexed occasionally and centrifuged for 10 min at 4 °C and 14,000 rpm. Supernatants were collected and protein concentrations were measured using the Bio-Rad Protein Assay Dye Reagent Concentrate (#5000006, Bio-Rad laboratories Inc.) according to the manufacturer’s protocol. For immunoprecipitation of Flag-tagged proteins, 0.5–1 mg of protein lysate was combined with 50 μl of anti-Flag® M2 affinity agarose gel (#A2220, Sigma-Aldrich) in 1 ml of lysis buffer. After 1 h rotation at + 4 °C, the agarose gel was washed four times with the lysis buffer. Samples were prepared by adding preheated 2x LSB, vortexing and heating for 5 min at + 95 °C. Coprecipitated proteins and lysate controls (50–100 μg) were analysed by Western blotting. For mass spectrometry, ~ 2.5 mg of protein was used for immunoprecipitation, after which CP subunits were separated by SDS-PAGE and stained by Page Blue.

For colocalization imaging, PLA and FLIM, PC-3 cells were transiently co-transfected on coverslips or left untransfected. After 24–48 h, samples were fixed at RT for 15 min with 4% PFA. For colocalization, cells were blocked in 10% BSA/PBS, followed by overnight incubation with the primary antibodies (Additional file 1: Table S4). On the following day, Alexa Fluor 488 goat anti-mouse (#A11001) or Alexa Fluor 647 goat anti-rabbit (#A21245) IgG secondary antibodies (Thermo Fisher Scientific) were used 1:1000 for 1 h at RT. During the secondary antibody staining, cells were simultaneously stained with DAPI (4′,6-diamidino-2-phenylindole dihydrochloride, #D9542, Sigma-Aldrich, 300 nM) and/or Alexa Fluor™ 546 Phalloidin (#A22283, Thermo Fisher Scientific, 150 nM) for visualization of the nuclei and/or actin cytoskeleton. For PIM inhibitor-treated, untransfected samples, Phalloidin–Atto 647 N (#65906, Sigma Aldrich, 100 nM) was used to stain actin filaments. Samples were imaged by the Zeiss LSM 780 confocal microscope with ZEN lite/Zen Black 2.3 software with 63x Zeiss C-Apochromat objective, numerical aperture: 1.2 (Carl Zeiss, Oberkochen, Germany) or the 3i CSU-W1 spinning disk confocal microscope with SlideBook 6 software, 63x Zeiss Plan-Apochromat objective, numerical aperture: 1.4 and Hamamatsu sCMOS Orca Flash4 v2 C11440-22CU camera (Intelligent, Imaging Innovations, Denver, CO, USA; Hamamatsu Photonics, Hamamatsu City, Shizuoka Pref., Japan). Colocalization was confirmed by Pearson’s correlation coefficiency test and Costes Threshold regression by ImageJ Coloc2 analysis. PLA was performed according to the manufacturer’s protocol using the Duolink® In Situ Orange Starter Kit Mouse/Rabbit (DUO92102, Sigma-Aldrich). PLA samples were imaged by the Nikon fluorescent microscope with NIS-Elements AR software (Nikon, Tokyo, Japan). Samples and figures were analysed and prepared using the respective microscopy software and ImageJ/Fiji. Samples for FLIM were directly mounted, while samples for the other assays were permeabilized in 0.1–0.2% Triton-X-100 for 15 min. FLIM was carried out as previously described [31, 33] using the Lambert Instruments LIFA FLIM system with the Carl Zeiss AxioImager microscope and LI-FLIM software (Lambert Instruments BV, Groningen, The Netherlands). All imaging was performed at room temperature. Actin filament length and number was manually measured in a double-blinded fashion using ImageJ/Fiji and calculating protrusions that were at least 3 μm long.

### Cell motility, attachment and viability assays

Cell migration, cell adhesion and cell viability assays were performed as described previously [29, 31]. For wound healing assays to measure cell migration, wounds were scratched by a 10 μl tip, after which they were imaged by light microscopy with Basler Microscopy Software 2.0 (Basler AG, Ahrensburg, Germany) and analysed by ImageJ/Fiji. Single cell tracking was performed at 37 °C, 5% CO_2_ by automated imaging with the Nikon Eclipse Ti2-E microscope and NIS-Elements AR 5.11.00 64-bit software with the 10x Nikon CFI Plan-Fluor objective, numerical aperture: 0.3., and the Hamamatsu sCMOS Orca Flash4.0 v3 C13440-20CU camera. Cell movement was tracked manually by ImageJ/Fiji from the .nd2 data files. Cell attachment was studied using the electrical impedance-based Roche xCelligence method with 15,000–25,000 cells plated per well. Cell viability was measured by the MTT assay [29]. Western blotting samples were prepared after the experiments to control for protein levels.

### Actin disassembly assays

Actin polymerization and disassembly assays as well as muscle actin production were performed as previously described [46]. 10 μM non-labelled (95%) and pyrene-labelled (5%) muscle actin were polymerized in a reaction mix containing 1 mM EGTA, 100 mM NaCl, 5 mM MgCl2. 0.2 mM ATP, 5 mM Tris-HCl pH 7.5, 0.2 mM DTT, 0.2 mM CaCl2. The solution was gently mixed and incubated first 15 min at RT and then 40 min at + 4 °C. Thereafter the disassembly assay was performed at RT in a reaction mix containing 1 mM EGTA, 100 mM NaCl, 5 mM MgCl2. 0.2 mM ATP, 5 mM Tris-HCl pH7.5, 1.2 mM DTT, 0.05 μM capping proteins produced in *E. coli* or the elution buffer (a negative control) and 4 μM of polymerized actin. The buffer and CPs were mixed gently by rotating a pipette tip in the tube and incubating for 5 min at RT. Thereafter, 4 μM vitamin D-binding protein (Human DBP, G8764, Sigma-Aldrich) was added to sequester actin monomers. Samples were carefully transferred into a cuvette, after which the decrease in pyrene-actin signal was measured by spectrophotometry (excitation 365 nm, emission 407 nm) for 4000 s. Average signal intensities were calculated by comparing each value to the first peak. The START time-point represents an average of values of 100 s time scale after the first peak. Similarly, average values from 100 s time scales were calculated for the other time-points as well: 1000 s (900–1000 s average), 2000 s (1900–2000 s average), 3000 s (2900–3000 s average) and 4000 s (3900–4000 s average).

### Statistical analysis and figure preparation

The student’s t-test was used to compare the difference between groups, while correlations were analysed by the Pearson’s correlation coefficiency. Significant differences (*P* < 0.05) are marked with asterisks in figures and supplementary files. Correlation coefficiencies (r [2]) were interpreted as very weak (0.00–0.19), weak (0.20–0.39), moderate (0.40–0.59), strong (0.60–0.79) or very strong (0.80–1.00). Error bars represent standard deviations. Corel Draw 2019 was used for figure preparation.

## Results

### Capping proteins are phosphorylated by PIM1 in prostate cancer cells

The initial aim of our study was to identify novel neuronal PIM substrates, as we have previously observed that *pim* family mRNAs are prominently expressed in the central nervous system [24] and that PIM kinases are active in neuron-like cells [25]. For this purpose, we used an in vitro phosphoproteomics-based method [38] and recombinant mouse Pim1 kinase to screen for target proteins from rat brain extracts. From this screen, we identified five potential Pim1 substrates: capping protein alpha 2 (Capza2), dihydropyrimidinase like 2 (Dpysl), enolase 1 (Eno1), endocytosis-associated protein 1 (Necap1) and prohibitin (Phb) (Additional file 2: Table S5). We next decided to focus our validation efforts on the capping protein (CP) family members, the phosphorylation of which could potentially allow PIM kinases to directly regulate actin dynamics and thereby cell motility.

**Fig. 1:**
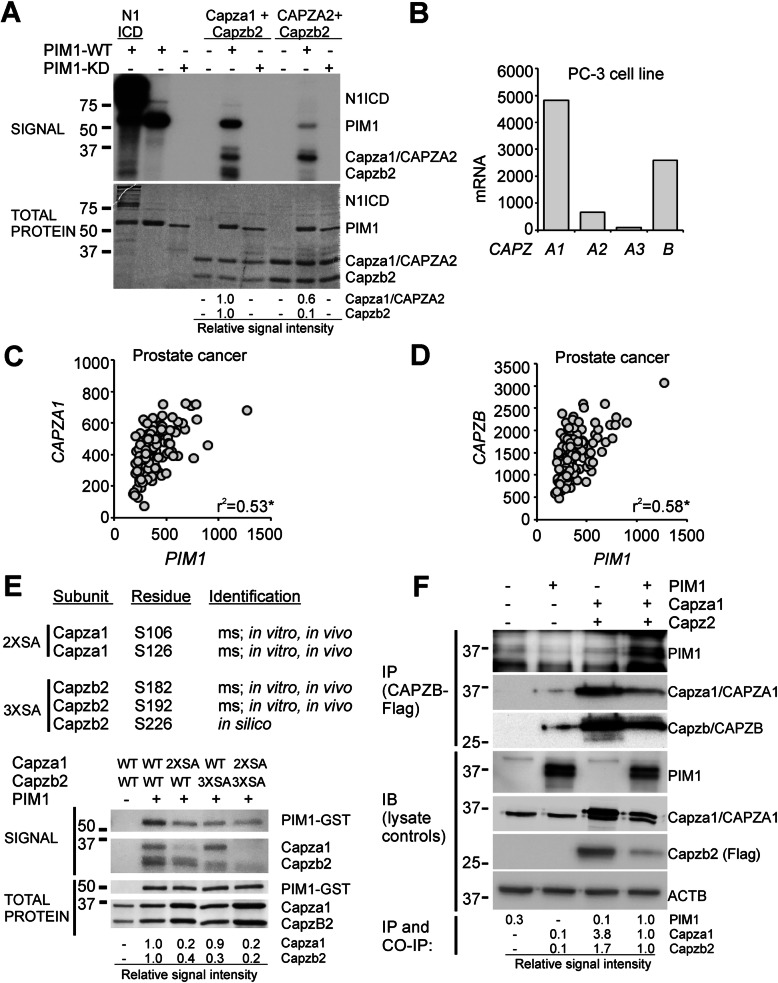
CPs are phosphorylated by and co-expressed with PIM1 in prostate cancer. **a** Radioactive in vitro kinase assays were performed by incubating human GST-tagged wild-type (WT) or kinase-deficient (KD) PIM1 with murine Notch1 intracellular domain (N1ICD, a positive control) or with murine His-Capza1 or human His-CAPZA2 together with murine Capzb2. Shown are results from one representative experiment out of three assays. Relative phosphorylation signal intensities as compared to loaded amounts of protein are shown under the blots. **b***CAPZ* mRNA levels expressed in human PC-3 prostate cancer cells were obtained from the IST Online™ database. **c**-**d** Pearson’s correlation coefficiencies were determined to *PIM* and *CAPZA1* or *CAPZB* mRNA levels in human prostate cancer patient samples obtained from the Betastasis database. Shown are correlations (r^2^) along with significance (P) values. **e** Mass spectrometry (ms) of in vitro and in vivo phosphorylated CP samples as well as in silico analysis were used to predict PIM kinase target sites. In vitro kinase assays were performed with GST-tagged PIM1 and either wild-type (WT) CP or serine to alanine (SA) mutants, where two alpha sites (2X; S106, S126) or three beta sites (3X; S182, S192, S226) had been mutated. Shown are representative figures with relative signal intensities. **f** Proteins interacting with Flag-Capzb2 were analysed from lysates of transiently transfected PC-3 cells. Shown are examples of immunoprecipitation (IP), immunoblotting (IB) and and co-immunoprecipitation (CO-IP). Note that the PIM1 antibody non-specifically detects also other proteins, most likely immunoglobulins

To analyse phosphorylation of CP subunits by human PIM1, radioactive in vitro kinase assays were performed targeting human CAPZA2 or mouse Capza1 together with mouse Capzb2. While these subunits had been selected for the study based on their availability, no species-specific differences were expected, as human and murine CPs are highly homologous to each other (amino acid identity for alpha 1 97%, alpha 2 98% and beta 2 100%) [44, 47]. The CPs were produced in bacteria as heterodimers, and incubated with wild-type (WT) or kinase-deficient (KD) human PIM1. As shown in Fig. 1a, WT, but not KD PIM1 was able to phosphorylate Capza1 and CAPZA2, although not as strongly as the Notch1 intracellular domain (ICD) used as a positive control. According to the relative signal intensities, Capza1 subunit was more efficiently phosphorylated by PIM1 than CAPZA2. Interestingly, however, Capzb2 was phosphorylated only when it formed a heterodimer with Capza1, but not with CAPZA2. These data validated the Capza1/b2 heterodimer as a prominent direct target of PIM1.

As we have previously shown that PIM kinases promote PC-3 prostate cancer cell motility [29–31, 36], we were interested in determining whether CPs are involved in this process. We therefore searched for *CP* mRNA expression levels from the IST Online™ database [42], according to which relatively high levels of *CAPZA1* and *CAPZB* had been observed in PC-3 cells as compared to *CAPZA2* or *CAPZA3* (Fig. 1b). For this reason, as well as for the absence of in vitro phosphorylation of Capzb2 with CAPZA2, our further studies focused on the functional consequences of phosphorylation of the Capza1/b2 heterodimer.

To evaluate whether CPs and PIM kinases are coexpressed in clinically relevant samples, we analysed the *PIM* and *CP* mRNA expression levels in prostate tumors of varying severity. According to data from the Betastasis database [43], both *CAPZA1* and *CAPZB* mRNA levels positively correlated with *PIM1* in primary prostate cancer patient samples (Fig. 1c-d). Positive correlations were also detected in metastatic tissues as well as between different *CP* and *PIM* isoforms (Additional file 2: Table S6). While *CAPZA1*, *CAPZA2* and *CAPZB,* but not *CAPZA3* levels correlated well with *PIM1* levels in all tumor tissues, similar correlations were also observed for PIM2 and PIM3 in the samples with high Gleason scores. By contrast, no correlations were seen in healthy prostate tissues. These data imply that PIMs and CPs are both present in the cancer tissues, suggesting that PIM-dependent phosphorylation of CPs can occur in this setting.

### Both CP subunits interact with and are phosphorylated by PIM1

To identify the PIM1 target residues, Capza1 and Capzb2 phosphorylation sites were analysed by mass spectrometry from in vitro kinase assays as well as from PC-3 cell co-immunoprecipitation assays. From those samples, several in vitro and in vivo sites were detected in both CP subunits (Fig. 1e, Additional file 2: Table S7). In addition, in silico analysis [48] of the Capzb2 amino acid sequence suggested that phosphorylation occurs at S226. Mass spectrometry also identified phosphorylation of Capzb2 at S2, but due to its close proximity to the N-terminus of the protein, it was expected to be an artefact and was not included in further studies. In addition, Capzb2 was found to be phosphorylated at T186 in vivo, but not in vitro, suggesting that it is targeted by a kinase other than PIM1. To validate the phosphorylation sites, the targeted serine residues were mutated to alanines (SA) and tested by in vitro kinase assays. Mutagenesis of the sites S106 and S126 in Capza1 and S182, S192 and S226 in Capzb2 decreased the phosphorylation signal efficiently and confirmed these sites as PIM1 target sites (Fig. 1e).

To analyse the interactions between PIM1 and the CP heterodimer, co-immunoprecipitation assays were performed. For this purpose, PC-3 cells were transiently transfected with different combinations of PIM1, Capza1 and Capzb2. When Flag-tagged Capzb2 was precipitated with Flag agarose, both Capza1 and PIM1 were co-precipitated with it (Fig. 1f), indicating that PIM1 and CPs physically interact with each other.

### CP subunits colocalize with PIM1 in the cytoplasm

To obtain information on CP localization in PC-3 prostate cancer cells, we analysed the subcellular distribution of endogenously expressed CAPZA1, CAPZA2 and CAPZB2 from fractionated cells by Western blotting. As controls for the CP-specific antibodies, PC-3 cells were transfected with His-tagged alpha subunits and Flag-tagged beta subunit, while lamin A/C and tubulin stainings were used as nuclear and cytoplasmic controls, respectively. Interestingly, both CAPZA1 and CAPZB proteins were mainly found from the cytoplasmic lysates, while CAPZA2 was mostly nuclear (Additional file 3: Fig. S1A-B).

**Fig. 2:**
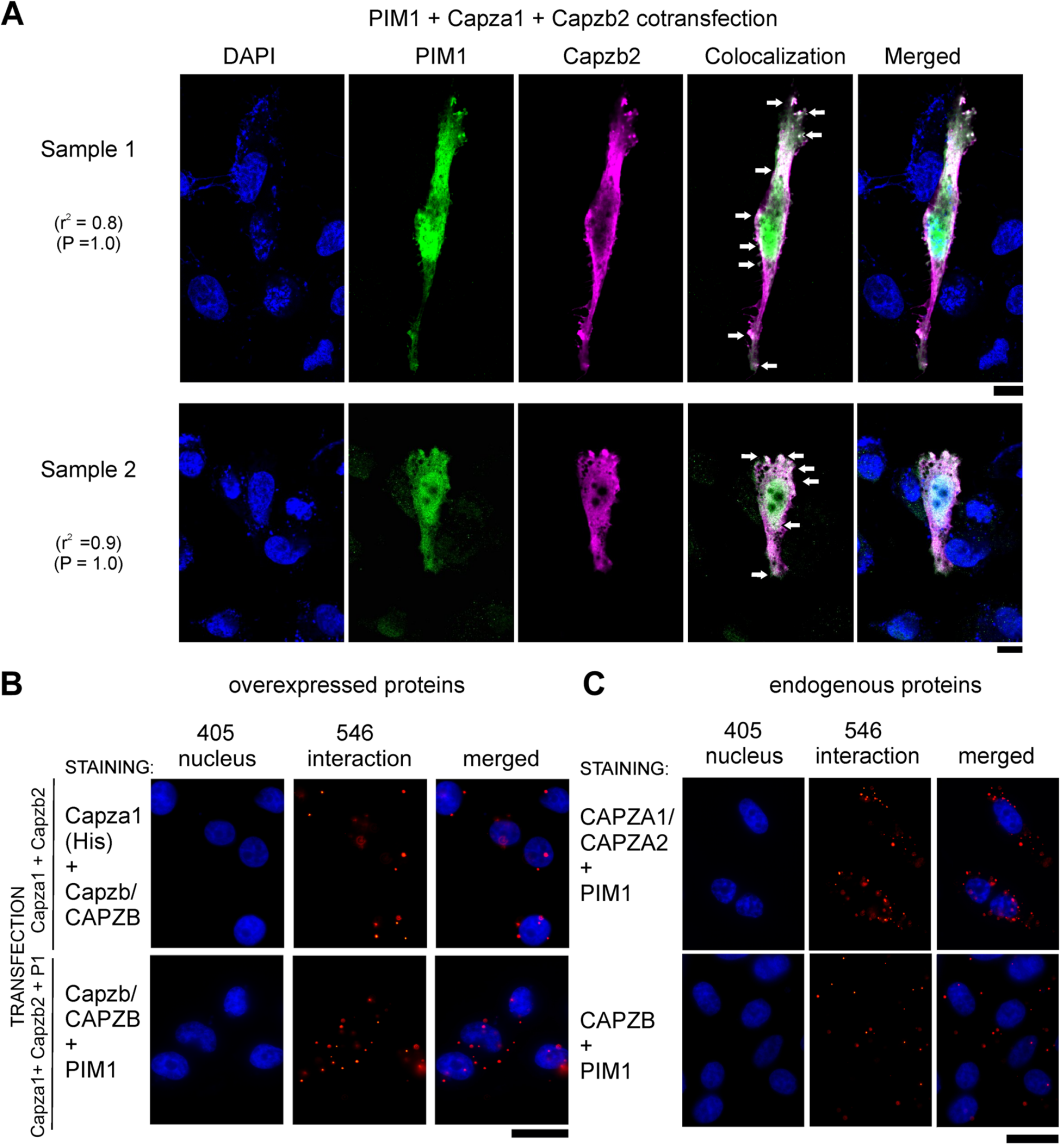
PIM1 interacts with the CP heterodimer in PC-3 prostate cancer cells. **a** PC-3 cells transiently overexpressing His-Capza1 and Flag-Capzb2 (CP) without or with PIM1 (P1) were stained for colocalization and **b-c** proximity ligation assays. DAPI staining was used in both assays to detect the nuclei. Shown are representative images as well as Pearson’s correlation values (r^2^) of colocalized pixels and their Costes significance (P) values. Scale bars represent 20 μm (**a**) or 25 μm (**b**-**c**)

Immunofluorescent stainings of transiently transfected PC-3 cells confirmed that His-tagged Capza1 colocalizes with Capzb2 in the cytoplasm (Additional file 3: Fig. S2A). Colocalization of PIM1 with the CP heterodimer was also observed in the cytoplasm at multiple positions (Fig. 2a). Negative controls for primary and secondary antibodies used in these assays are shown in the Additional file 3: Fig. S2B. In addition, we analysed colocalization of PIM1 with either ectopically or endogenously expressed alpha subunits. While in Western blotting, the CAPZA1 and CAPZA2 antibodies detected both subunits equally well (Additional file 3: Fig. S2C), only the CAPZA2 antibody worked in the immunofluorescent stainings. However, most likely it was unable to penetrate into the nuclei, as only cytoplasmic signals were observed that most likely were derived from the CAPZA1 subunit (Additional file 3: Fig. S2D).

Interactions between PIM1 and CPs were then analysed by proximity ligation assay (PLA), which confirmed interactions between overexpressed Capza1, Capzb2 and PIM1 (Fig. 2b), but revealed interactions also between endogenously expressed proteins (Fig. 2c). Additional controls for PLA assays with statistical analyses are shown in Additional file 3: Fig. S3A-B.

Additional confirmatory data on cellular interactions were obtained by fluorescence-lifetime imaging microscopy (FLIM). For that purpose, Capzb2 was N-terminally tagged with GFP, and expressed either alone or together with His-tagged Capza1. When these plasmids were co-overexpressed in PC-3 cells with RFP-tagged PIM1, the lifetime of GFP fluorescence was reduced, indicating the presence of physical interactions between PIM1 and Capzb2 (Additional file 3: Fig. S4A-B). However, addition of the large fluorescent tag increased the nuclear localization of Capzb2 (Additional file 3: Fig. S4C-D), which is why the more cytoplasmic Flag-tagged version of Capzb2 was chosen for further functional assays.

### CP phosphorylation promotes PC-3 prostate cancer cell motility

As we have previously performed wound healing assays to demonstrate the pro-migratory effects of PIM kinases in PC-3 cells [29], we now wanted to analyse the role of CP phosphorylation in this setting. For this purpose, we used a dual expression vector to simultaneously overexpress different combinations of wild-type (WT) and phosphomutant forms of both CP subunits. For phosphodeficient mutants, PIM-targeted serine residues were mutated to alanines (SA) and for phosphomimicking mutants, they were mutated to glutamic acids (SE). From here on, the SA or SE mutants are referred to with the mutated residue number or with double (2X; S106 and S126 in Capza1, or S182 and S192 in Capzb2) or triple (3X; S182, S192, S226 in Capzb2) mutations. To facilitate interpretation of the results, the graphs have been colour-coded: non-transfected or mock-transfected samples (grey), wild-type CPs (black), Capza1 SA/SE mutants (orange/yellow), Capzb2 SA/SE mutants (purple/blue), and combined Capza1/b2 SA/SE mutants (dark green/light green).

**Fig. 3:**
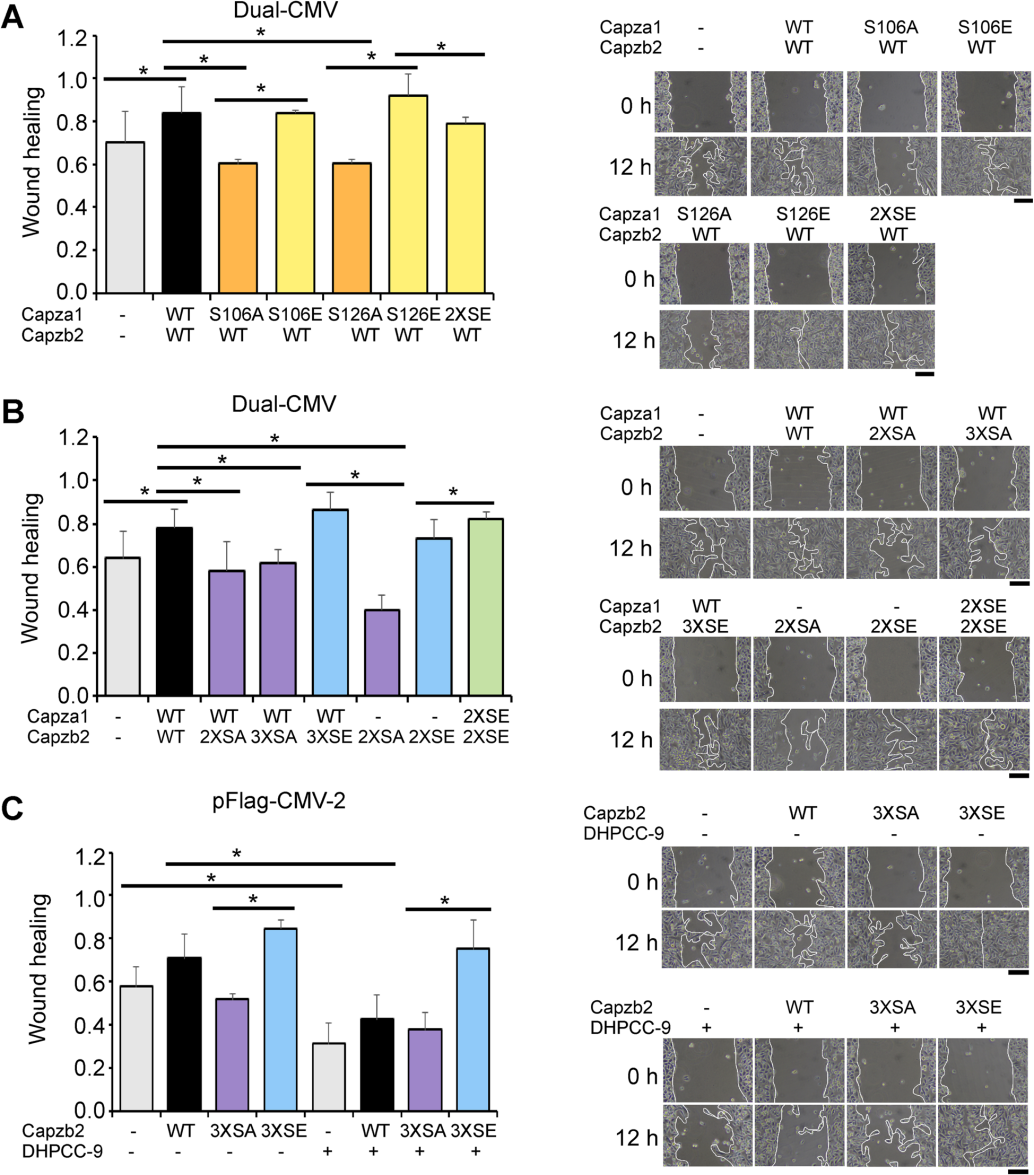
CP phosphorylation promotes PC-3 cell migration. **a**-**b** Wound healing assays were performed with PC-3 cells transiently overexpressing Capza1/b2 wild-type (WT) proteins and/or phosphodeficient (SA) or phosphomimicking (SE), single, double (2X) or triple (3X) mutants. **c** Similar assays were performed with cells that overexpressed only Capzb2 variants and were treated with DMSO (0.1%) or 10 μM PIM inhibitor DHPCC-9. Shown are averages from at least three independent experiments with three parallel samples along with representative images. Wound edges are lined in white. Scale bars represent 20 μm. Expression plasmid names are shown on top of the graph bars

In the wound healing assays, both wild-type CPs and the phosphomimicking mutants promoted PC-3 cell migration as compared to controls or the phosphodeficient mutants (Fig. 3a-b). In the case of the alpha subunit, quite similar results were obtained after mutagenesis of either one of the PIM target sites. The equivalent effects of the double and triple mutants of Capzb2 indicated that lack of the two identified in vivo sites was sufficient to inhibit cell migration. Furthermore, the 2XSE mutant of Capzb2 was sufficient to support wound healing also in the absence of co-overexpressed Capza1, while the 2XSA mutant of Capzb2 alone reduced cell motility. This implies that the ectopically expressed beta subunits dimerize with the endogenous alpha subunits to regulate cell movements. As the pan-PIM inhibitor DHPCC-9 not only efficiently and selectively inhibits catalytic activities of all three PIM family kinases, but also slows down PC-3 cell migration [29], we tested its effects in cells transfected with Flag-tagged Capzb2. In the DMSO-treated control samples, Capzb2 WT and 3XSE increased cell motility in an expected fashion, while DHPCC-9 treatment resulted in significantly decreased migration in all other samples except for the sample expressing the 3XSE mutant (Fig. 3c). Thus, the ability of this phosphomimicking mutant to rescue the anti-migratory effects of the PIM inhibitor strongly suggests that phosphorylation of CPs is essential for PIM-induced changes in cell motility.

To confirm that the above data were not due to differences in CP expression or cell viability, these parameters were analysed. No major changes were detected in WT versus mutant CP expression levels (Additional file 3: Fig. S5A-D). However, the levels of Capzb2 SA or SE mutants were reduced, when they were overexpressed alone without Capza1 (Additional file 3: Fig. S5C). MTT assays in turn showed that the CP mutants did not have major effects on cell viability, while the PIM inhibitor DHPCC-9 slightly decreased it in the transfected cells (Additional file 3: Fig. S6A-C).

### Phosphorylation of CPs regulates formation of actin protrusions

**Fig. 4:**
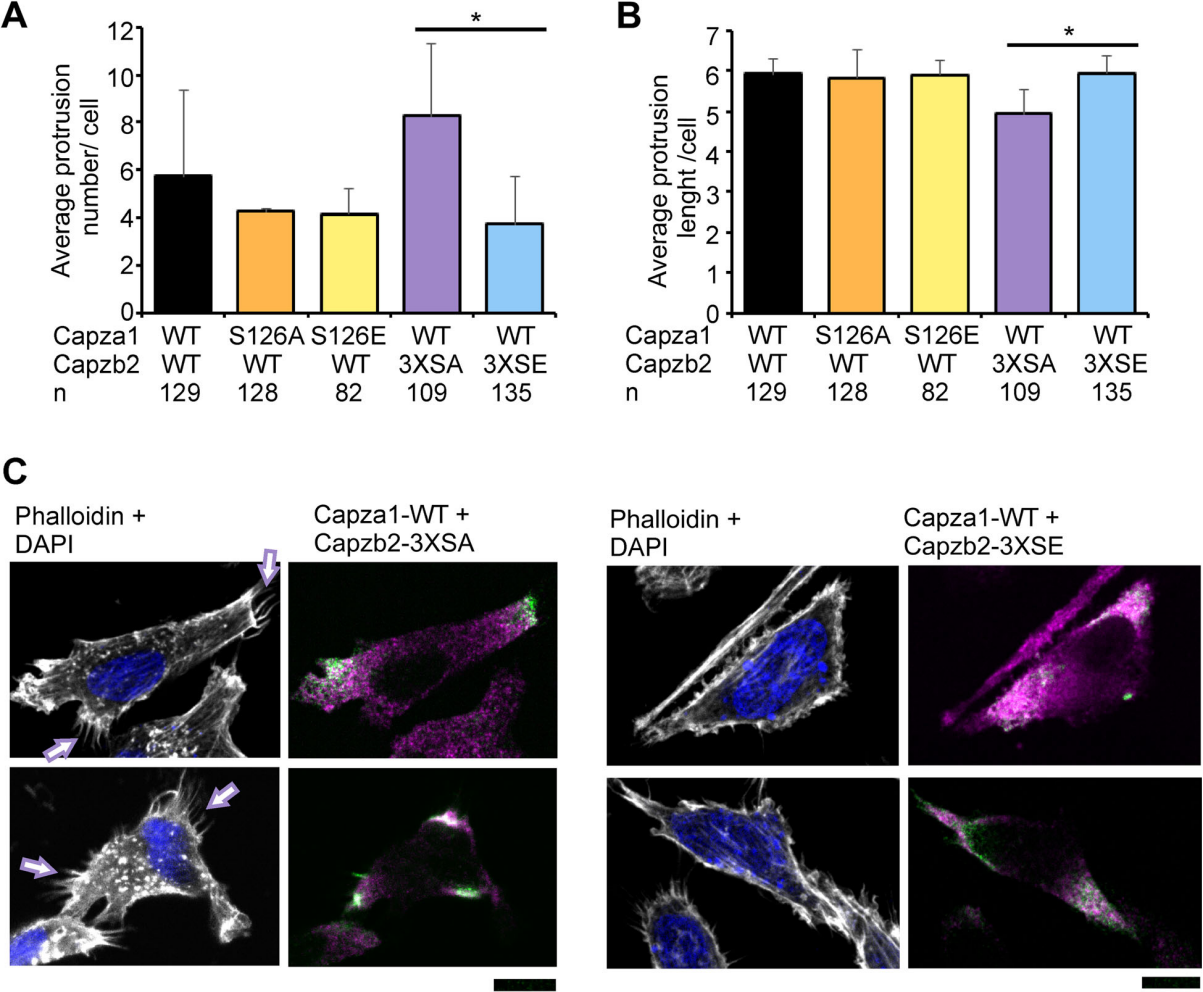
CP dephosphorylation increases actin protrusion numbers at cell edges. **a**-**c** PC-3 cells plated on coverslips were transiently transfected to overexpress wild-type (WT) Capza1 and Capzb2 or their phosphodeficient (SA) or phosphomimicking (SE) mutants. Actin filaments were stained by phalloidin, and actin protrusion numbers and lengths were measured. Shown are averages from at least three individual samples along with representative images for the Capzb2 phosphomutants. The increase in actin protrusion numbers in SA samples is pointed out by arrows. Scale bars represent 10 μm

As CPs regulate actin polymerization and have previously been reported to regulate actin protrusion formation at the cell edges [49], we wanted to measure the effects of CP phosphorylation on the formation of actin filopodia at the leading edge and retraction fibers at the lagging edge. For this purpose, actin filaments were stained by phalloidin and imaged in transiently transfected PC-3 cells. Overexpression of the phosphodeficient (3XSA) Capzb2 led to significantly increased protrusion numbers, while the protrusions were also slightly shorter than in the other samples (Fig. 4a-c, Additional file 3: Fig. S7). We counted both leading edge filopodia and lagging edge retraction fibers, provided they were over 3 μm long. By contrast, both single phosphomutants of Capza1 as well as the triple phosphomimicking (3XSE) mutant of Capzb2 slightly reduced the protrusion number as compared to wild-type, but did not affect their length.

**Fig. 5:**
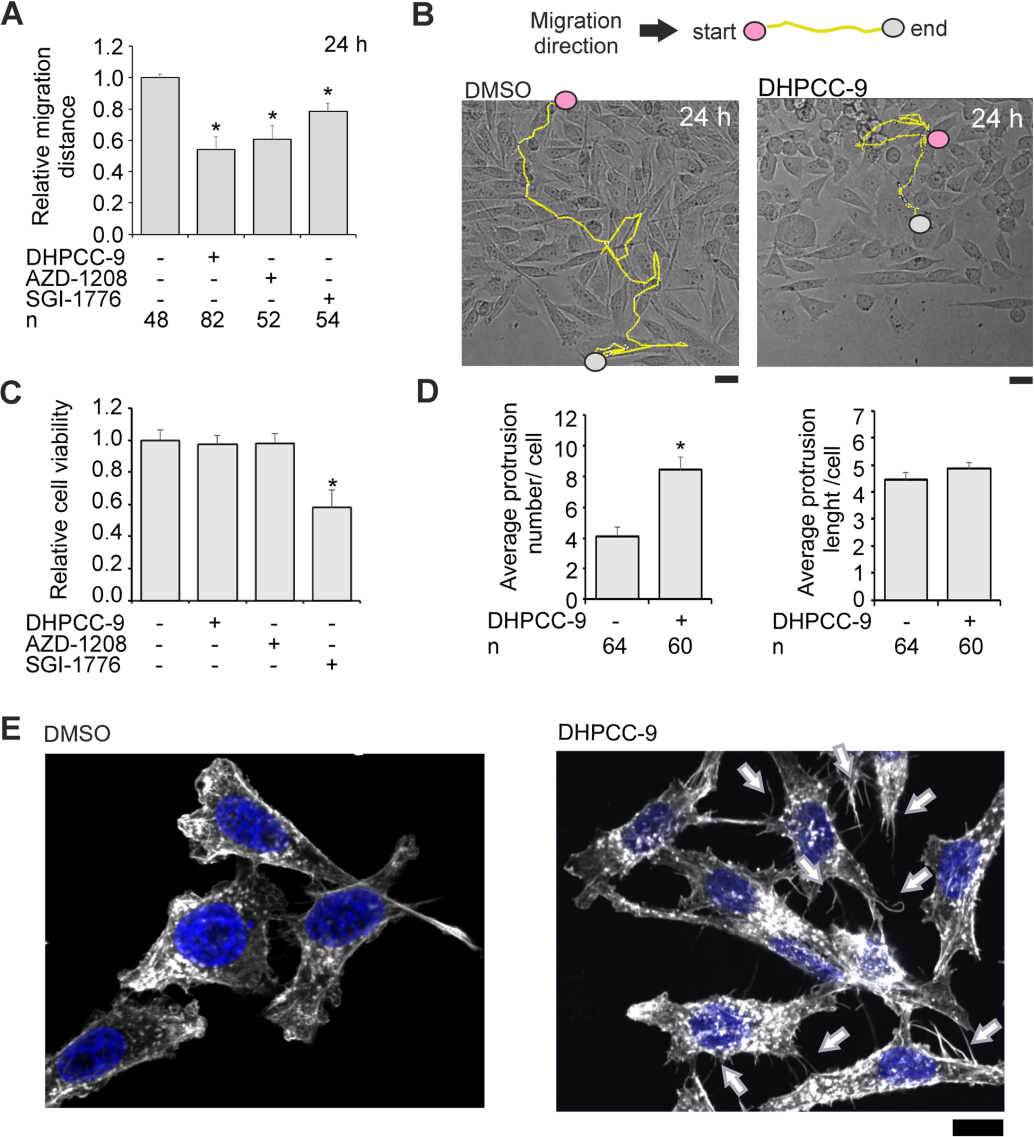
PIM inhibition increases the number of actin protrusions at cell edges. PC-3 cells were treated for 24 h with 10 μM DHPCC-9, AZD-1208 or SGI-1776. Single cell tracking was performed to visualize the migration route and distance after PIM inhibition. **a** Shown are averages of indicated numbers (n) of cells along with **b** representative images of cells treated with DMSO or DHPCC-9. Scale bars represent 20 μm. **c** MTT assays were used to determine the relative viability of PC-3 cells after 24 h treatments with PIM inhibitors. Shown are averages from three independent experiments with three parallel samples. **d** PC-3 cells were plated on coverslips and treated with DMSO or DHPCC-9 for 24 h, after which actin filaments were stained by phalloidin, and the number of actin protrusions and their length were measured. Shown are averages of indicated numbers (n) of cells along with **e** representative images for actin protrusions. Scale bars represent 10 μm. The increase in actin protrusion numbers in DHPCC-9-treated samples is pointed out by arrows

When the effects of three structurally distinct PIM inhibitors (DHPCC-9, AZD-1208 and SGI-1776) were compared by single cell tracking in wound healing assays, all inhibitors reduced PC-3 cell migration, but DHPCC-9 was most efficient (Fig. 5a, b). Data from MTT assays indicated that both DHPCC-9 and AZD-1208 were well tolerated within the 24 h follow-up period, whereas SGI-1776 showed significant cytotoxicity (Fig. 5c). When DHPCC-9-treated cells were stained with phalloidin, the number of actin protrusions was dramatically increased, but without major effects on their length (Fig. 5d-e). Altogether, these data suggest that CP phosphorylation by PIM kinases restricts the ability of cells to form actin protrusions at cell edges.

### CP phosphorylation increases cell adhesion

**Fig. 6:**
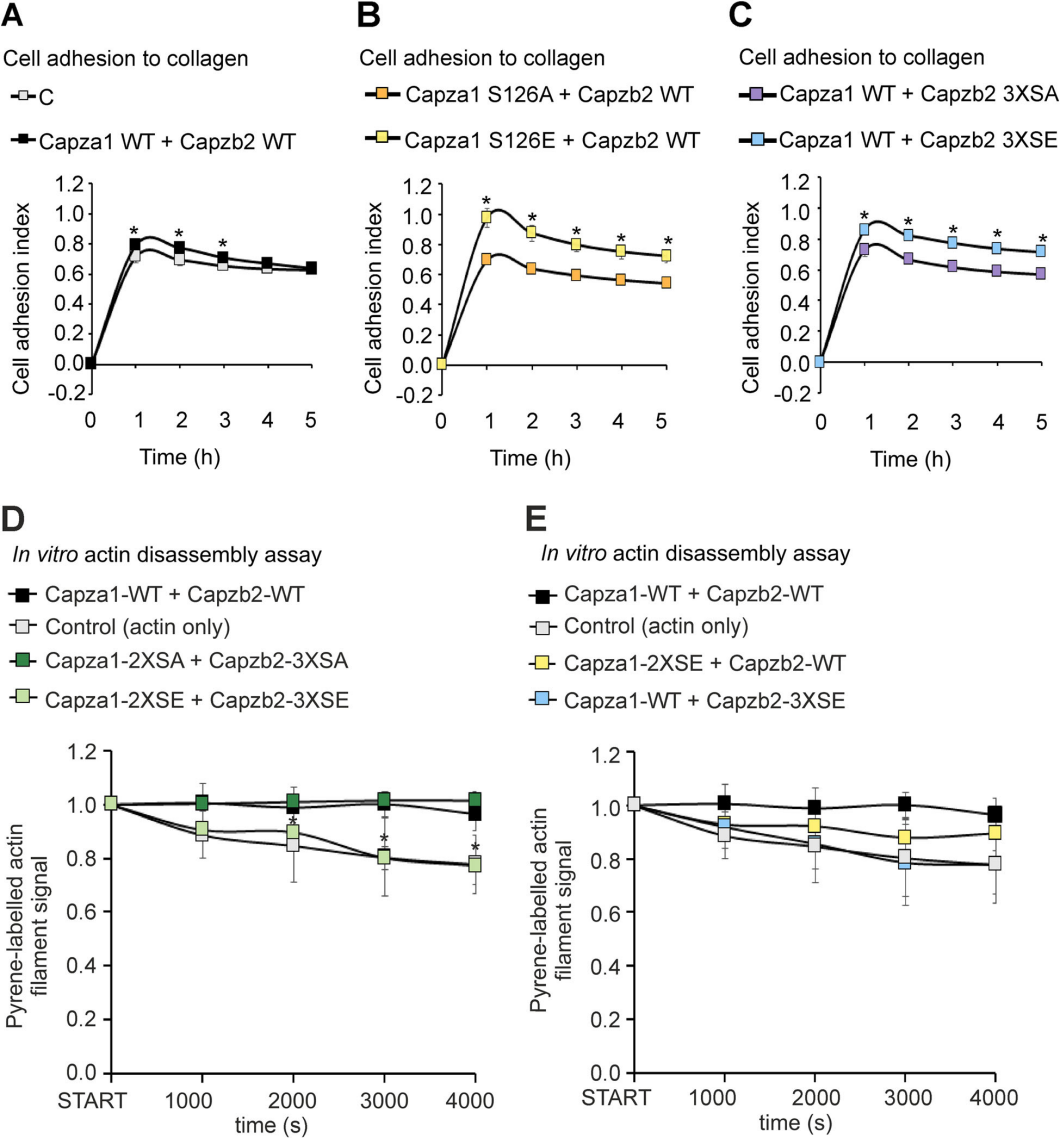
CP phosphorylation increases cell adhesion and actin disassembly. **a**-**c** PC-3 cells were transiently transfected with wild-type (WT) Capza1 and Capzb2 or their phosphodeficient (SA) or phosphomimicking (SE) mutants. Cell adhesion to collagen was recorded according to the electrical impedance. Similar experiments were performed at least three times with three parallel samples, while shown are representative results from one experiment with three parallel samples. The statistical analyses were performed pairwise for each graph. **d**-**e** Actin polymerization was performed by combining muscle actin with 5% pyrene-labelled actin. Thereafter actin disassembly was followed in vitro with or without addition of bacterially produced CPs. Actin re-assembly was prevented by vitamin D-binding protein. Shown are average results from two to four independent experiments with different combinations of samples. The statistical analyses were performed by comparing to the CP wild-type control (Capza1-WT + Capzb2-WT)

We have previously observed that PIM inhibition by DHPCC-9 inhibits cell adhesion to collagen and fibronectin matrices [31]. To analyse the effects of CP phosphorylation on cell adhesion, we used xCelligence to measure PC-3 cell attachment according to the electrical impedance (resistance to alternating current). Cell adhesion to collagen was slightly enhanced by overexpression of WT Capza1 and Capzb2 (Fig. 6a), and was further enhanced by the phosphomimicking mutants (9–24% increase as compared to the wild-type protein), while the phosphodeficient mutants had an opposite effect (8–16% decrease as compared to the wild-type protein) (Fig. 6b-c). By contrast, all cells adhered equally poorly to poly-L-lysine, which was used as a negative control surface (Additional file 3: Fig. S8A-C).

### PIM-mediated CP phosphorylation increases actin disassembly

To mechanistically analyse the effects of PIM-mediated phosphorylation on CP actin capping activity, actin disassembly assays were performed with or without wild-type or mutant CPs. After actin polymerization, the decrease in pyrene-labelled actin fluorescence was followed up for more than an hour (4000 s). Vitamin D-binding protein was added to prevent actin re-assembly, as it has been shown to sequester actin monomers, allowing us to follow actin depolymerization and CP effects on the process [50, 51]. While actin by itself slowly started to disassemble over time, wild-type CPs prevented actin disassembly, but the phosphomimicking mutants did not (Fig. 6d-e). Here it should be noted that the bacterially produced wild-type protein was non-phosphorylated, and was thereby expected to behave similarly to the phosphodeficient mutant. These results indicate that phosphorylation indeed interferes with the actin capping activity.

### Phosphorylation of CPs also promote the motility of DU-145 prostate cancer cells

Finally, we wanted to compare the effects of PIM kinases on the motility of other prostate cancer cell lines. For this purpose, we used qPCR to measure relative *PIM* mRNA levels in PC-3, DU-145 and LNCAP cell lines (Additional file 3: Fig. S9A-C). *PIM1* mRNA expression was lower in LNCAP cells as compared to the others, while no significant differences were detected in *PIM2* and *PIM3* mRNA levels between the cell lines. When we performed wound healing assays, we found that DU-145 cells migrated faster than PC-3 cells, and that also their motility could be reduced by a treatment with the DHPCC-9 PIM inhibitor (Additional file 3: Fig. S9D-E). By contrast, the migration rate of LNCAP cells was very low and not affected by PIM inhibition (Additional file 3: Fig. S9F).

When we compared CP expression in different prostate cancer cell lines by Western blotting, we observed slightly lower levels of CAPZA1 in PC-3 cells as compared to the other lines, while CAPZB levels were highest in DU-145 cells (Additional file 3: Fig. S10A). To analyse the role of CP phosphorylation in the rapidly migrating DU-145 cells, wound healing assays were carried out with cells that had been transiently transfected to overexpress wild-type Capza1 with wild-type or mutant Capzb2. No major changes were detected between the expression levels of wild-type and phosphomutant proteins (Additional file 3: Fig. S10B). Similarly to the data from PIM inhibitor-treated cells, migration of DU-145 cells was affected by CP phosphorylation, but in a less pronounced fashion (Additional file 3: Fig. S10C-D). When adhesion of DU-145 cells was analysed, PIM inhibition by DHPCC-9 reduced adhesion to collagen in a similar manner as observed in PC-3 cells (Additional file 3: Fig. S10E). Furthermore, transient overexpression of wild-type Capzb2 or its phosphomimicking (3XSE) mutant significantly promoted cell adhesion as compared to control cells or those overexpressing the phosphodeficient (3XSA) mutant (Additional file 3: Fig. S10F-G). Data from MTT assays indicated that even though DHPCC-9 slightly reduced viability of LNCAP cells, it had no effects in DU-145 cells, the viability of which was also only marginally affected by CP overexpression (Additional file 3: Fig. S11A-C).

## Discussion

The oncogenic PIM kinases support tumor growth as well as cancer cell adhesion, migration, invasion and the formation of metastases through multiple signalling pathways [23]. Here we have identified capping proteins as novel PIM substrates and demonstrate that PIM-dependent phosphorylation of the CP heterodimers inhibits their actin capping activity and thereby enhances actin dynamics and prostate cancer cell motility. To investigate the impact of phosphorylation, the CP subunits or their phosphomimicking (SE) or phosphodeficient (SA) mutants were simultaneously overexpressed using a dual plasmid. To reduce potential off-target effects, which have previously been associated with high expression levels [52, 53], all overexpression experiments were initiated 12 h after transfection.

In our study, we observed that both subunits of the Capza1/b2 heterodimer are phosphorylated by PIM1, while in the other heterodimer formed by human CAPZA2 and mouse Capzb2, only CAPZA2 was phosphorylated. This cannot be explained by species-specific differences, as the beta subunits in mouse and human are identical (Additional file 3: Fig. S12). Furthermore, also the two alpha 1 phosphorylation sites and their surrounding sequences are fully conserved between mouse and human proteins (Additional file 3: Fig. S12). Based on mRNA expression levels [42], the CAPZA1/B2 heterodimer is more prominently expressed than the CAPZA2/B2 in the PC-3 prostate cancer cells, which were used in most of our cellular assays. However, according to our Western blotting data, both CAPZA1 and CAPZA2 proteins are expressed in PC-3 cells, but have distinct subcellular localizations. Alpha 1 is cytoplasmic, but alpha 2 mostly nuclear, hinting at distinct functions for the CAPZA1/B2 and CAPZA2/B2 heterodimers. Based on our imaging and immunoprecipitation data, PIM1 colocalizes and interacts with the CP heterodimer mainly in the cytoplasmic regions, and promotes both cell adhesion and migration in a phosphorylation-dependent fashion. On the other hand, phosphorylation of CAPZA2 was not studied in more detail and it may therefore play an as of yet unknown role in the regulation of e.g. nuclear actin dynamics in prostate cancer cells.

Previously, capping protein activity has been reported to be inhibited by multiple factors, while no activators have been discovered. Proteins such as formins and Ena/VASP proteins regulate CP function indirectly, while myotrophin, phosphoinositides and CARMIL proteins inhibit CPs directly by binding to them [6]. Very little is known about post-translational modification of CPs, except that Capza1 can be phosphorylated by the casein kinase CK-2 at S9 [22], and CAPZB1 acetylated at K199 and phosphorylated by PKCε at S204 [14]. All these modifications reduce capping activity. Here we have shown that both CP alpha and beta subunits are phosphorylated by PIM1: Capza1 at S106 and S126, and Capzb2 at S182, S192 and S226. Phosphorylation decreases the ability of CPs to protect actin filament ends and thereby increases dynamical changes in actin filament organization. This promotes the shortening or elongation of filamentous actin. Here, in the phosphodeficient samples, the filament dynamics is blocked, and increased numbers of slightly shortened filaments are seen. The continuous changes in actin organization are known to be needed for proper cell adhesion and migration [54]. As expected, we observed increases in both cell adhesion and motility by overexpressing the phosphomimicking mutants. The balance of CP activity is expected to be crucial for proper actin dynamics and cell shape formation. For instance, CP beta silencing has been shown to increase the number of filopodia on cell edges [49], while complete silencing or overexpressing CPs leads to embryonic death [53].

All other PIM phosphorylation sites were identified from cellular samples, except for S226 in Capzb2. However, phosphorylation of this residue may be context-dependent, as it has previously been observed to be phosphorylated in colorectal cancer samples [48]. It should also be noted that some of the PIM phosphorylation sites, as well as the additional T186 site of Capzb2, may be targeted by other kinases as well. Furthermore, the PIM consensus sequences (K/R-K/R-R-K/R-L-S/T-X [55], K/R-K/R-K/R-X-S/T-X [56] and R-X-R-H-X-S [57]) do not completely match with the CP target sites, which is also the case with several other previously identified PIM kinase substrates [36, 58–61]. Therefore, it may be worth re-evaluating the PIM1 consensus sequence in the future.

Our data may also have clinical implications. Here we show positive correlations between *PIM1* and either *CAPZA1*, *CAPZA2* or *CAPZB* mRNAs in prostate cancer samples with different Gleason grades, but not in the healthy control tissues. Also, *PIM2* and *PIM3* mRNA expression levels correlate with those of CPs in more advanced tumors. Thus, the similar expression patterns of PIM kinases and CPs at different cancer stages suggest that their interactions may affect prostate cancer progression via phosphorylation-dependent regulation of the capping activity. This is supported by our comparative studies with additional prostate cancer cell lines. Interestingly, the hormone-independent prostate cancer cell lines PC-3 and DU-145 behave quite similarly, while no major phosphorylation-dependent changes are detected in the androgen-dependent LNCAP cells with relatively low PIM1 levels. Thus, the higher PIM1 levels and increased activity of PIM-mediated signalling pathways in PC-3 and DU-145 cells may be connected to their increased motility as compared to LNCAP cells.

From a therapeutic perspective, it is crucial to know the downstream targets of PIM kinases and to be able to estimate the on- and off-target effects of PIM inhibitors. According to our results, PIM inhibition is expected to increase CP activity, resulting in decreased cancer cell migration. However, it would be important to analyse the effects of PIM kinases and their inhibitors on other CP subunits to confirm that PIM inhibition does not cause any unexpected changes in healthy tissues, such as muscle, testis or the nervous system, from where we originally identified the Capza2 subunit as a neuronal substrate for PIM1.

## Conclusions

To summarize, CP alpha and beta subunits have previously been reported to behave in an opposite fashion [17–21], leading to contradictory interpretations of the role CPs play in cancer progression. Here we have demonstrated that it is essential to simultaneously analyse the effects of both CP subunits as a heterodimer, which is the functional unit protecting the ends of actin filaments. We have shown that phosphorylation of the alpha 1 and beta 2 subunits by PIM1 reduces the actin capping activity of the CP heterodimer, resulting in increased prostate cancer cell motility. This is also the first study linking PIM kinases directly to the regulation of actin dynamics, highlighting the importance of PIM family members in enhancing motility and metastatic behaviour of cancer cells.

## Supplementary information

**Additional file 1.** Additional protocols tables. Tables S1-S4 show detailed data related to the methods of the study.

**Additional file 2.** Additional results tables. Tables S5-S7 show additional data related to the results shown in the main figures.

**Additional file 3 **Additional results figures*.* Figures S1-S12 show additional data related to the results shown in the main figures.

## Data Availability

The databases used for in silico analysis are the following: PhosphoMotif Finder (www.hprd.org/PhosphoMotif_finder/), the PhosphoSitePlus® database (phosphosite.org), IST Online™ database (ist.medisapiens.com), Betastasis database (betastasis.com; gene expression barblot and Taylor et al. dataset), the Basic Local Alignment Search Tool (blast.ncbi.nlm.nih.gov/Blast.cgi), the National Center for Biotechnology Information (www.ncbi.nlm.nih.gov/) and UniProt Knowledgebase (www.uniprot.org/help/uniprotkb). Plasmid backbones are the following: pGEM-T-Easy (#A1360, Promega, https://fi.promega.com), pRFSDuet-1 (#71341, Merck Millipore; www.merckmillipore.com), GST vectors (GE Healthcare Life Sciences; www.fishersci.com), PSF-CMV-CMV-SBFI-UB-PURO and pFlag-CMVTM-2 (#OGS597 and # E7033, Sigma-Aldrich; www.sigmaaldrich.com), pEGFP-C1 (Clontech laboratories Inc.; www.addgene.org), pcDNATM3.1/V5-His (#V81020, Thermo Fisher Scientific, www.thermofisher.com), Tag-RFP-N (#FP142, Evrogen; evrogen.com).

## Abbreviations

2X: double mutant (S106 and S126 in Capza1 or S182 and S192 in Capzb2)
3X: triple mutant (S182, S192, S226 in Capzb2)
CP: capping protein
DHPCC-9: 1,10-dihydropyrrolo [2,3-a]carbazole-3-carbaldehyde, compound 9 (PIM inhibitor)
Dual-CMV: PSF-CMV-CMV-SBFI-UB-PURO - DUAL CMV
Duet: pRFSDuet-1
ICD: intracellular domain (of Notch receptor)
KD: kinase-deficient
MCS: multiple cloning site
pcDNA-PIM1: pcDNA™3.1/PIM1-V5-His
SA: serine (S) to alanine (A) phosphomutant (phosphodeficient)
SE: serine (S) to glutamic acid (E) phosphomutant (phosphomimicking)
WT: wild-type
