# Supplementary material for: PIM1 accelerates prostate cancer cell motility by phosphorylating actin capping proteins
Source: Cell Commun Signal. 2020 Aug 8;18:121. doi: 10.1186/s12964-020-00618-6 (PMC7414696; doi:10.1186/s12964-020-00618-6)
Supplement: Supplementary file 2 — Additional file 1. Additional protocols tables. Tables S1-S4 show detailed data related to the methods of the study. [file 12964_2020_618_MOESM2_ESM.pdf]

Table S1 – Primers used for cloning and sequencing

Shortenings: pRFSDuet-1 (Duet), pGEM-T-Easy (Easy), pGEX-6P-3 (GST), PSF-CMV-CMV-SBFI-UB-PURO - DUAL CMV (Dual) and pFlag-CMV-2 (Flag); multiple cloning site (MCS), cloning primer (c), sequencing primer(s), bacterial expression vector (be), bacterial cloning vector (bc) or mammalian expression vector (me).

Vector	Insert	Primer	Primer sequence 5´- 3´
Duet (be)	Capza1 (MCS 1)	Forward (s)	GGATCTCGACGCTCTCCCT
Duet (be)	Capza1 (MCS 1)	Reverse (s)	GATTATGCGGCCGTGTACAA
Duet (be)	Capzb2 (MCS 2)	Forward (s)	TTGTACACGGCCGCATAATC
Duet (be)	Capzb2 (MCS 2)	Reverse (s)	GCTAGTTATTGCTCAGCGG
Easy (bc)	CAPZA2	Forward (c)	AGGCTTATGGCGGATCTGGAGGAGCAG
Easy (bc)	CAPZA2	Reverse (c)	GCGGCCGCTCATGCATTCTGCATCTCTTT
Easy (bc)	Capzb2	Foward (c)	GCCGCCCCATGAGCGATCAGCAG
Easy (bc)	Capzb2	Reverse (s)	TCTTCAACACTGCTGCTTTCTCTTCAAGGC
Easy (bc)	CAPZA2	Forward (s)	GTAATACGACTCACTATAGGGC
GST (be)	Capzb2	Forward (s)	ATAGCATGGCCTTTGCAGGGCTG
Dual (me)	Capza1/ CAPZA2	Forward (s)	GGATCTCGACGCTCTCCCT
Dual (me)	Capza1/ CAPZA2	Reverse (s)	GATTATGCGGCCGTGTACAA
Dual (me)	Capzb2	Forward (s)	TTGTACACGGCCGCATAATC
Dual (me)	Capzb2	Reverse (s)	GCTAGTTATTGCTCAGCGG
To Dual from Flag (me)	Capzb2	Forward (s)	GGCATGTCGACATGGACTACAAAGAC
To Dual from Flag (me)	Capzb2	Reverse (s)	CCTCTAGAGTCGATCGACTGGTACC

Table S2 – Plasmid design

Shortenings: pRFSDuet-1 (Duet), pGEM-T-Easy (Easy), pGEX-6P-3 (GST), PSF-CMV-CMV-SBFI-UB-PURO - DUAL CMV (Dual), pFlag-CMV-2 (Flag) and pEGFP-C1 (GFP); multiple cloning site (MCS), digestion prior to insert (Digestion 1) and digestion after the insert (Digestion2), blunt end generation with Klenow fragment or digestion (italics), disruption of digestion site (overlining), insert (i), vector (v) and N-terminal (N-).

**Vector**	**Insert**	**Digestion 1**	**Digestion 2**	**MCS 1 or 2**	**(N-)Tag**
Duet	Capza1	NcoI	~~BamHI~~	**1**: Capza1<br>**2**: Capzb2	His
Duet	Capzb2	NdeI	~~BglII~~	**1**: Capza1 or CAPZA2<br>**2**: Capzb2	-
Duet	CAPZA2	EcoRI	NotI	**1**: Capza2<br>**2**: Capzb2	His
Easy	CAPZA2	EcoRI	SpeI	**1**	-
Easy	Capzb2	EcoRI	SpeI	**1**	-
GST	Capzb2	EcoRI	EcoRI	**1**	GST
Dual	Capza1	NcoI	*i: EcoRI or NotI*<br>*v: EcoRV*	**1**: Capza1<br>**2**: Capzb2	His
Dual	CAPZA2	NcoI	*i: NotI*<br>*v: EcoRV*	**1**: CAPZA2<br>**2**: Capzb2	His
Flag	Capzb2	EcoRI	EcoRI	**1**	Flag
Dual	Capzb2	SalI	SpeI	**1**: Capza1 or CAPZA2<br>**2**: Capzb2	Flag
GFP	Capzb2	EcoRI	EcoRI	**1**	GFP
Dual	GFP-<br>Capzb2	*i: NheI,*<br>*v: PciI*	*i: KpnI*<br>*v: SpeI*	**1**: Capza1<br>**2**: Capzb2	GFP

Table S3 – Primers for mutagenesis

Amino acid residues of wild-type or mutant proteins: serine (S), alanine (A) and glutamic acid (E).

Protein	Mutagenesis	Primer type	Primer sequence
Capza1	S106>A	Forward	GGAAAGAAGCAGCCGACCCGCAGCCAGAGG
Capza1	S106>A	Reverse	CCTCTGGCTGCGGGTCGGCTGCTTCTTTCC
Capza1	S106>E	Forward	GGAAAGAAGCAGAGGACCCGCAGCCAGAGG
Capza1	S106>E	Reverse	CCTCTGGCTGCGGGTCCTCTGCTTCTTTCC
Capza1	S126>A	Forward	GGAGGGAGTCGTGTGATGCTGCGCTGAGAGCC
Capza1	S126>A	Reverse	GGCTCTCAGCGCAGCATCACACGACTCCCTCC
Capza1	S126>E	Forward	GGGAGTCGTGTGATGAGGCGCTGAGAGCCTATG
Capza1	S126>E	Reverse	CATAGGCTCTCAGCGCCTCATCACACGACTCCC
Capzb2	S182>A	Forward	CTATGGCTGCAAACTAACAAGGCTGGCTCGGGCAC
Capzb2	S182>A	Reverse	GTGCCCGAGCCAGCCTTGTTAGTTTGCAGCCATAG
Capzb2	S182>E	Forward	GCAAACCAACAAAGAAGGCTCGGGCACCATGAAC
Capzb2	S182>E	Reverse	GTTCATGGTGCCCGAGCCTTCTTTGTTGGTTTGC
Capzb2	S192>A	Forward	GAACCTGGGAGGCGCACTAACGAGACAGATGG
Capzb2	S192>A	Reverse	CCATCTGTCTCGTTAGTGCGCCTCCCAGGTTC
Capzb2	S192>E	Forward	GAACCTGGGAGGCGAACTAACGAGACAGATGG
Capzb2	S192>E	Reverse	CCATCTGTCTCGTTAGTTCGCCTCCCAGGTTC
Capzb2	S226>A	Forward	GAAAACAAAATCCGGGCCACGCTGAATGAGATCTAC
Capzb2	S226>A	Reverse	GTAGATCTCATTCAGCGTGGCCCGGATTTTGTTTTC
Capzb2	S226>E	Forward	GGACATGGAAAACAAAATCCGAGAGACGCTGAATGAG
Capzb2	S226>E	Reverse	CTCATTCAGCGTCTCTCGGATTTTGTTTTCCATGTC

Table S4 – Primary antibodies

Samples were stained overnight at +4°C in rotation in PBS or TBS Tween-20 according to manufacturer´s protocols for Western blotting (WB) or in a humidified chamber for immunofluorescence (IF).

**Protein or tag**	**Company**	**Product number**	**Dilution for WB**	**Dilution for IF**
PIM1	Merck	MABC553	1:500	1:500
Capza1	Abcam	ab166892	1:1000	-
CAZPA1/A2	Abcam	ab175378	1:1000	1:500
Capzb2	Sigma	HPA031531	1:500	1:500
His	Thermo Fisher Scientific	MAI_21315	1:2000	1:500
Flag	Sigma	F3165	1:500	1:500
Tubulin	Cell Signaling Technology	#86298	1:1000	-
Lamin A/C	Cell Signaling Technology	#4777	1:1000	-
ACTB	Cell Signaling Technology	#4970, #3700	1:1000	-
