# Supplementary material for: PIM1 accelerates prostate cancer cell motility by phosphorylating actin capping proteins
Source: Cell Commun Signal. 2020 Aug 8;18:121. doi: 10.1186/s12964-020-00618-6 (PMC7414696; doi:10.1186/s12964-020-00618-6)
Supplement: Supplementary file 3 — Additional file 2. Additional results tables. Tables S5-S7 show additional data related to the results shown in the main figures. [file 12964_2020_618_MOESM3_ESM.pdf]

Table S5 – Identification of potential Pim1 substrates by mass spectrometry

Rat proteins phosphorylated by murine Pim1 in an *in vitro* phosphoproteomics screen were identified by mass spectrometry. The recognized peptides are shown alongside their UniProt identifiers.

UniProt entry code	Rat protein	Identified peptides
Q3T1K5	Capza2	FIIHAPPGEFNEVFNDVR; EATDPRPYEAENAIESWR; NFWNGR
P47942	Dpysl2	MVIPGGIDVHTR; FQMPDQGMTSADDFFQGTK; GTVVYGEPITASLGTDGSH; DNFTLIPEGTNGTEER; VFNLYPR
P04764	Eno1	AAVPSGASTGIYEALELR; GVPLYR; LAMQEFMILPVGASSFR; AGYTDQVVIGMDVAASEFYR; FTASAGIQVVGDDLTVTNPK
P69682	Necap1	ASDWKLDQPDWTGR; LDQPDWTGR; VSGELFAQAPVEQYPGIAVETVTD; SAFIGIGFTDR; SAFIGIGFTDRGDAFDFNVSLQDH; GDAFDFNVSLQDHFK
P67779	Phb	FDAGELITQR; DLQNVNITLR; VLPSITTEILK; IYTSIGEDYDER; KLEAAEDIAYQLSR; AAELIANSLATAGDGLIELR; AATFGLILDDVSLTHLTFGK; FGLALAVAGGVVNSALYNVDAGHR

Table S6 – Correlation of PIM and CAPZ levels in prostate tissues

Pearson´s correlation coefficiencies and their statistical significance (*) in human samples.

Gene	*PIM1*	*PIM2*	*PIM3*	Tissue type
CAPZA1	0,53*	0,28	0,26	**Primary tumor**
CAPZA2	0,59*	0,27	0,20	
CAPZA3	-0,08	0,03	0,03	
CAPZB	0,58*	0,36	0,39	
CAPZA1	0,58*	-0,13	0,22	**Metastasis**
CAPZA2	0,62*	-0,03	0,28	
CAPZA3	-0,46*	0,16	-0,23	
CAPZB	0,52*	0,16	0,50*	
CAPZA1	0,69*	0,27	0,17	**Gleason score <7**
CAPZA2	0,70*	0,25	0,22	
CAPZA3	-0,23	-0,15	-0,07	
CAPZB	0,63*	0,34	0,36	
CAPZA1	0,42*	0,18	0,22	**Gleason score 7**
CAPZA2	0,51*	0,18	0,14	
CAPZA3	0,04	0,22	0,12	
CAPZB	0,56*	0,33	0,41*	
CAPZA1	0,72*	0,60*	0,47*	**Gleason score >7**
CAPZA2	0,82*	0,66*	0,32*	
CAPZA3	-0,17	-0,02	-0,02	
CAPZB	0,76*	0,58*	0,43*	
CAPZA1	0,02	-0,20	0,16	**Healthy prostate tissue**
CAPZA2	0,20	-0,23	0,16	
CAPZA3	0,03	0,05	-0,05	
CAPZB	0,18	-0,12	0,10	

Table S7 – Identification of CAPZ phosphorylation sites

Phosphorylation of Capza1 and Capzb2 by PIM1 was analysed by *in vitro* kinase assays. For cellular *in vivo* analyses, Capza1 and Capzb2 were co-overexpressed with PIM1 in PC-3 prostate cancer cells, and Capza1 was co-immunoprecipitated with Capzb2. In both cases, proteins separated by SDS-PAGE were cut out, and subjected to trypsin digestion and mass spectrometry analysis with TiO_2 enrichment. Shown are protein names, codes and identified phosphopeptides along with other observed modifications.

**Protein (Uniprot code)**	**Residue**	**Peptide**	**Modifications**	***in vitro***	***in vivo***
Capza1 (P47753)	S106	KEASDPQPEDVDGGLK	S4-Phospho	x	x
Capza1 (P47753)	S126	ESCDSALR	C3-Carbamidomethyl, S5-Phospho	x	x
Capzb2 (P47757)	S2	SDQQLDCALDLMR	S1-Phospho, C7-Carbamidomethyl, M12-Oxidation	x	-
Capzb2 (P47757)	S182	SGSGTMNLGGSLTR	S1-Phospho, M6-Oxidation	x	x
Capzb2 (P47757)	T186	SGSGTMNLGGSLTR	T5-Phospho	-	x
Capzb2 (P47757)	S192	SGSGTMNLGGSLTR	M6-Oxidation, S11-Phospho	x	x
