# Supplementary material for: PIM1 accelerates prostate cancer cell motility by phosphorylating actin capping proteins
Source: Cell Commun Signal. 2020 Aug 8;18:121. doi: 10.1186/s12964-020-00618-6 (PMC7414696; doi:10.1186/s12964-020-00618-6)
Supplement: Supplementary file 4 — Additional file 3 Additional results figures. Figures S1-S12 show additional data related to the results shown in the main figures. [file 12964_2020_618_MOESM4_ESM.pdf]

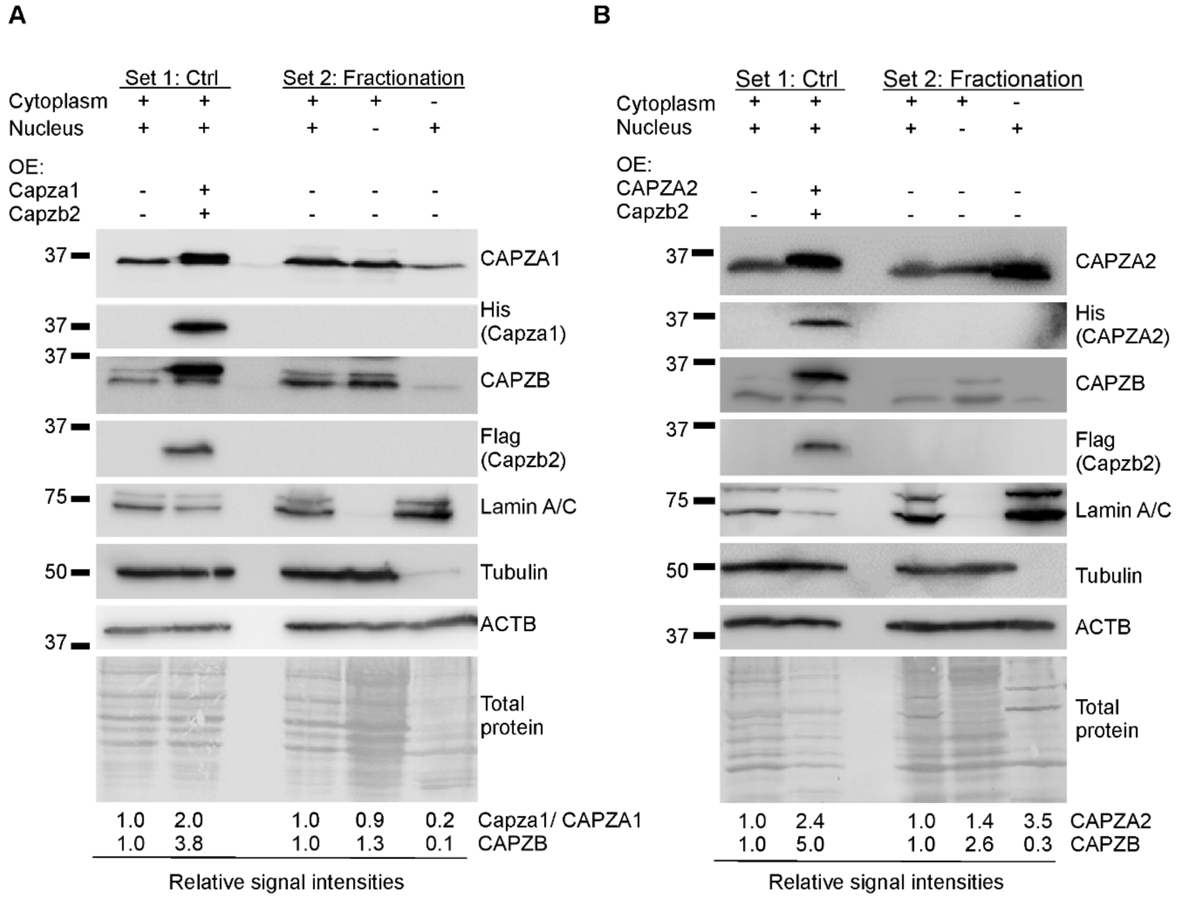

Figure S1 – CAPZA1 and CAPZB2 are expressed mainly in the cell cytoplasm, unlike CAPZA2

(A-B) Untransfected PC-3 cells were fractionated into nuclear and cytoplasmic fractions and stained with antibodies against CP proteins, while lysates from PC-3 cells overexpressing His- and Flag-tagged Capza1/b2 **(A)** or CAPZA2/b2 **(B)** were used as positive controls. Lamin A/C, Tubulin, ACTB and total protein staining were used as localization and loading controls.

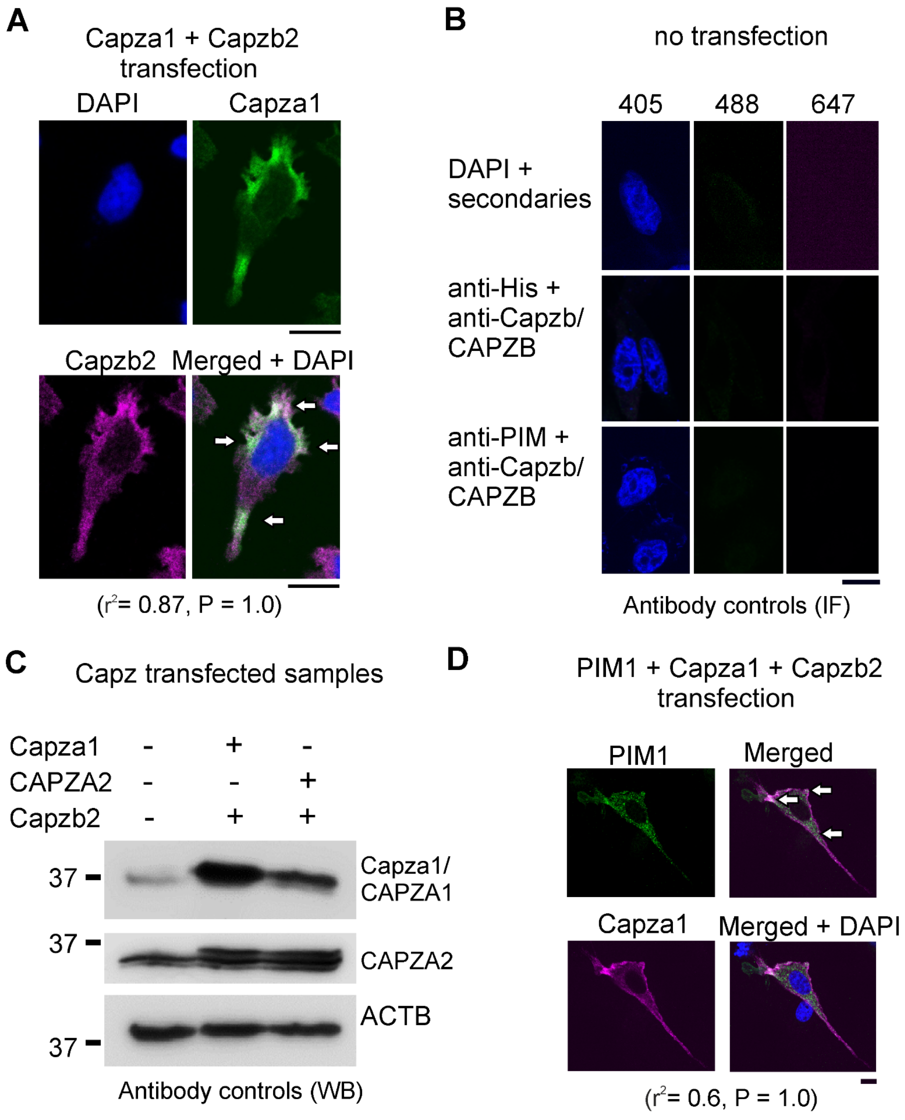

Figure S2 – Immunofluorescence controls and additional colocalization assays

(A) PC-3 cells transiently overexpressing His-Capza1 and Flag-Capzb2 were stained with His and Capzb antibodies. **(B)** Untransfected cells were used as controls for immunofluorescence, where adjusted signal intensity and laser power for the overexpression were too low to detect the endogenous proteins. **(C)** Capza/CAPZA antibody specificity was tested with transfected PC-3 cell samples. **(D)** Transfected PC-3 cells were stained with Capza2 and PIM1 antibodies to measure the co-localization between PIM1 and Capza1. Colocalization is shown in white in the merged images and pointed-out by arrows. Scale bars represent 20 µm (A-B) or 10 µm (D). Shown are also Pearson´s correlations (r^2) of colocalized pixels and their Costes significance (P) values.

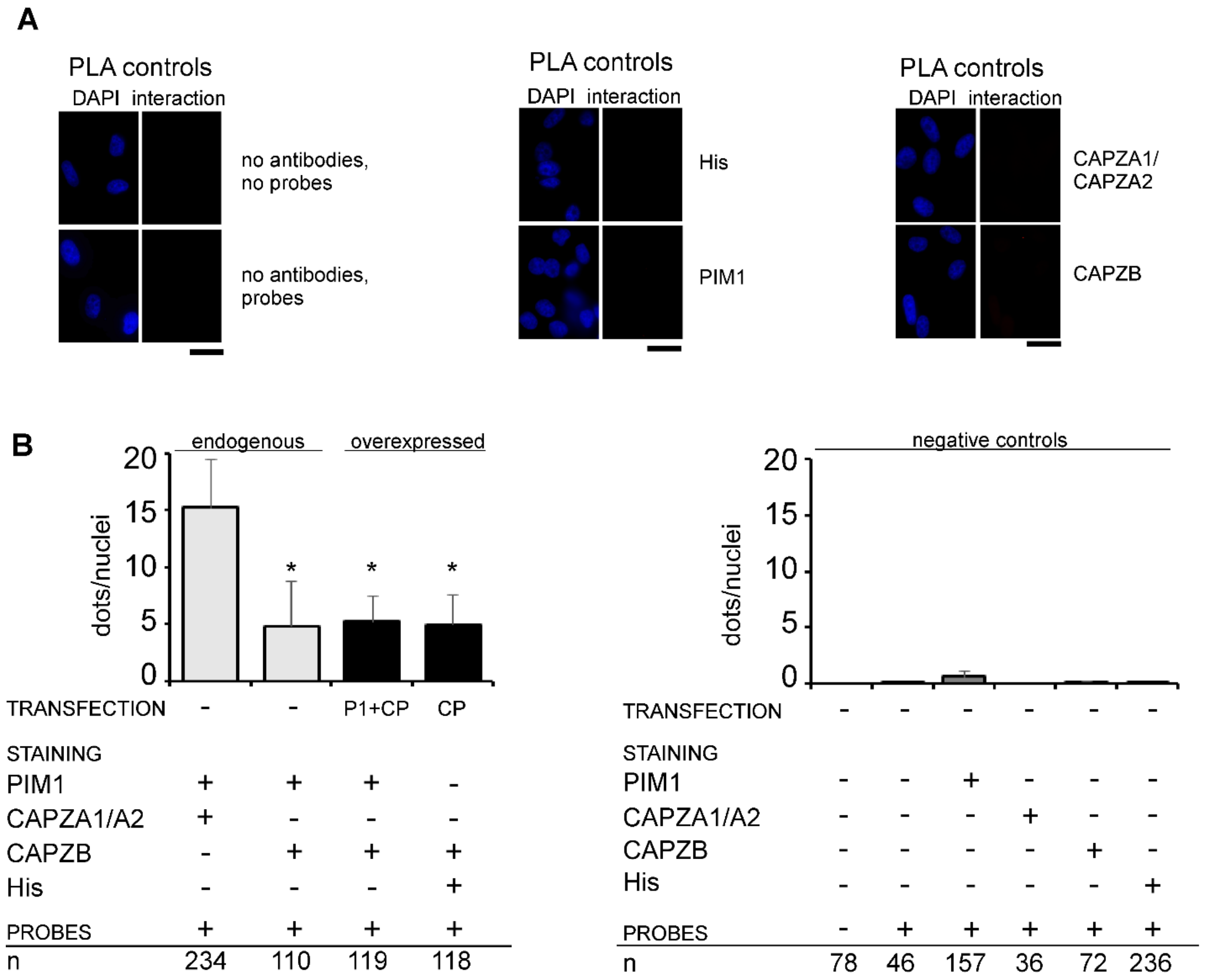

Figure S3 – Controls and analysis of proximity ligation assays

(A) Untransfected PC-3 cells were used as negative controls for the indicated antibodies in proximity ligation assays (PLA). Antibodies are shown in the right. DAPI staining was used to visualize nuclei. Scale bars represent 25 µm. **(B)** Summary of PLA data with analysed cell numbers (n). Shown are stainings for endogenous proteins or ectopically overexpressed PIM1 (P1) and/ or Capza1 + Capzb2 (CP). As negative controls, we used both samples with probes and without probes to confirm that we did not have any background signals.

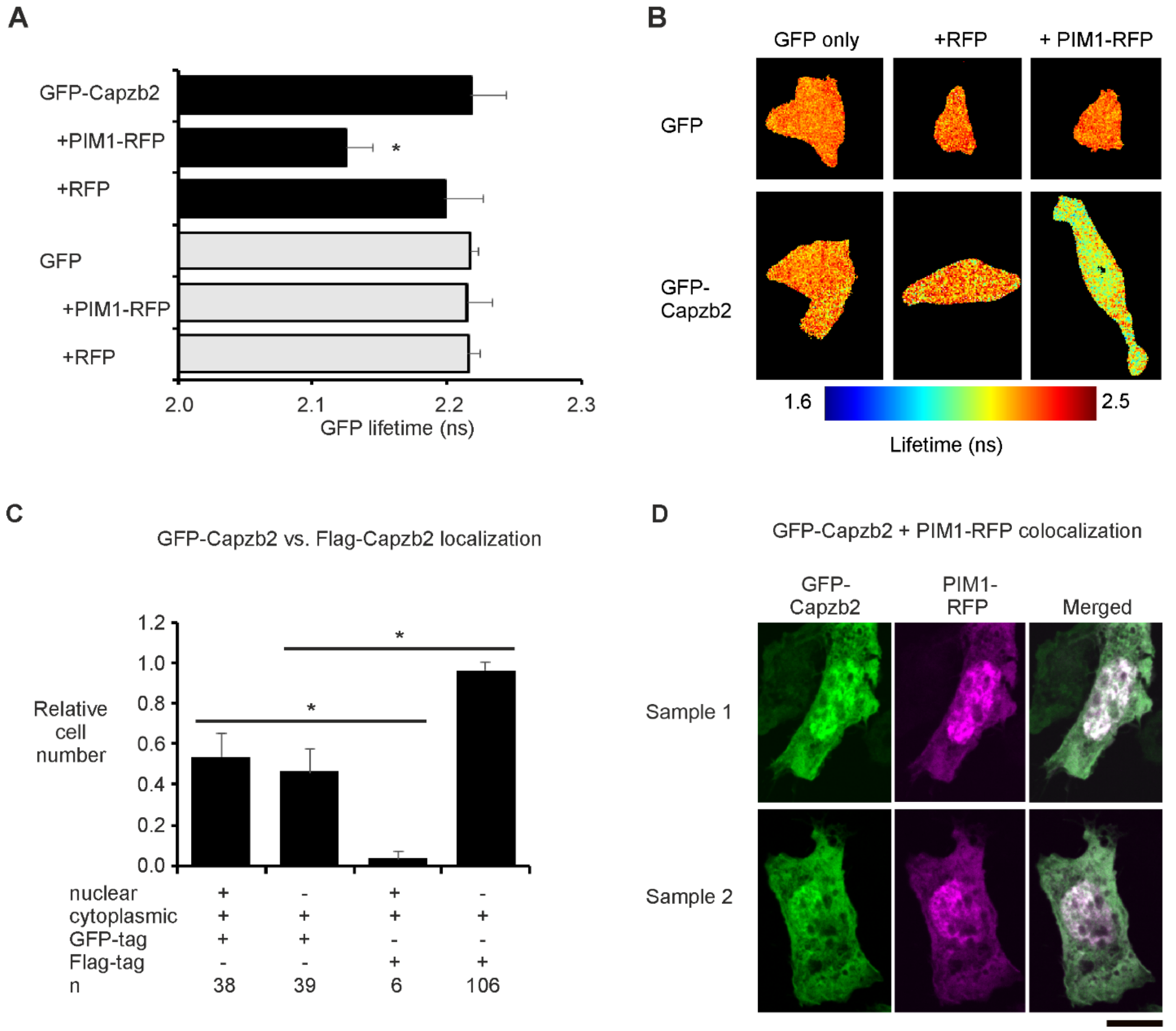

Figure S4 – GFP-Capzb2 and PIM1-RFP interact and colocalize in cells

(A) PC-3 cells were plated on coverslips and transfected with empty vectors or vectors overexpressing GFP-tagged mouse Capzb2 or RFP-tagged human PIM1 followed by fixation and fluorescence-lifetime imaging. Shown are average GFP lifetimes along with analysed cell numbers inside the bars. Lifetimes are combined from two individual experiments with parallel samples, with either GFP-tagged Capzb2 alone or in combination with His-tagged Capza1. **(B)** Representative figures show the decreased GFP lifetime due to protein-protein interaction. **(C)** The localization of GFP- versus Flag-tagged Capzb2 was analysed from control samples without PIM1 overexpression. Both GFP- and Flag-tagged Capzb2 proteins were co-overexpressed with the wild-type Capza1 subunit. **(D)** Representative figures show colocalization of GFP-Capzb2 and PIM1-RFP in both nuclei and cytoplasm. For visualization, the RFP red color has been turned into magenta. Scale bar represents 10 µm.

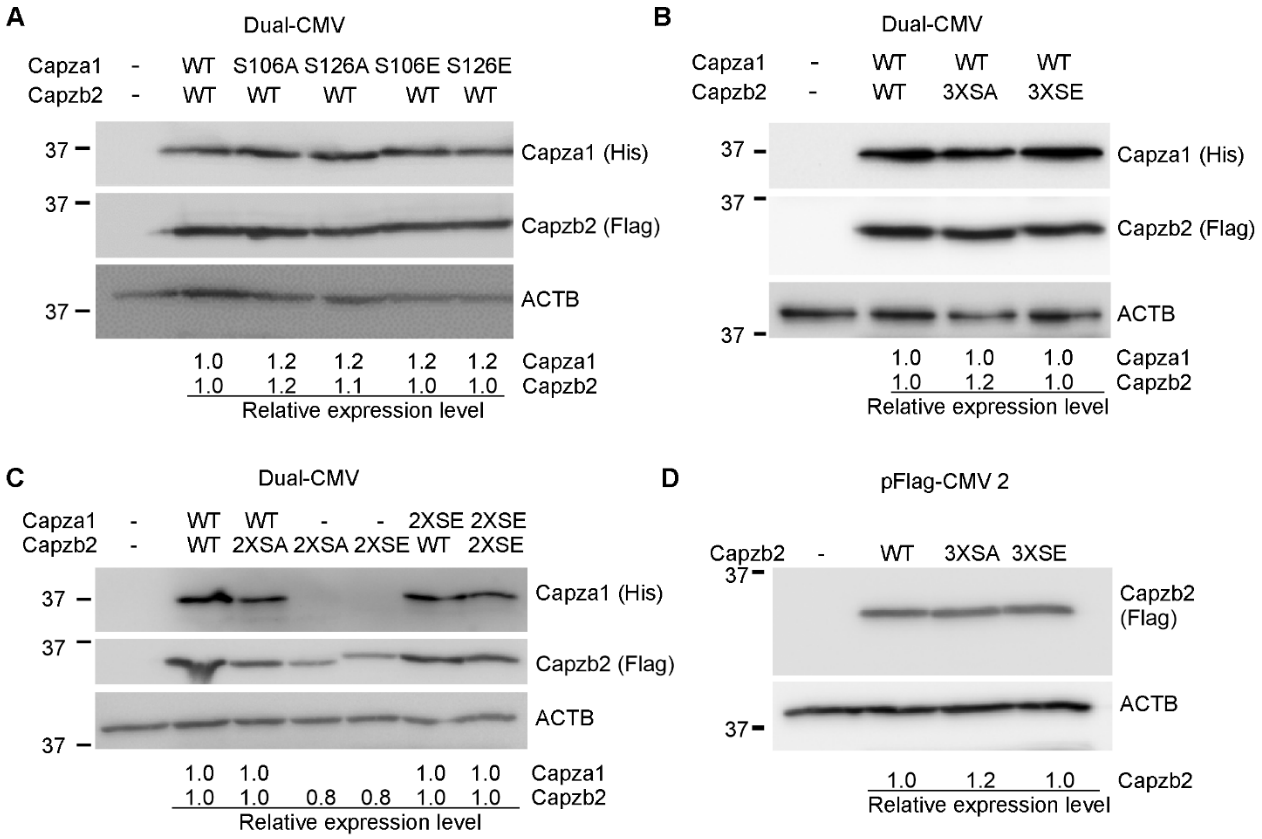

Figure S5 – CP mutants are expressed similarly to the wild-type proteins

(A-D) Wild-type (WT) Capza1 and Capzb2 or phosphomimicking (SE) or phosphodeficient (SA) single, double (2X) or triple (3X) phosphomutants were transiently overexpressed in PC-3 cells from plasmids indicated above the blots. Samples were prepared for Western blotting after wound healing assays, and overexpression was confirmed by targeting CP tags with antibodies.

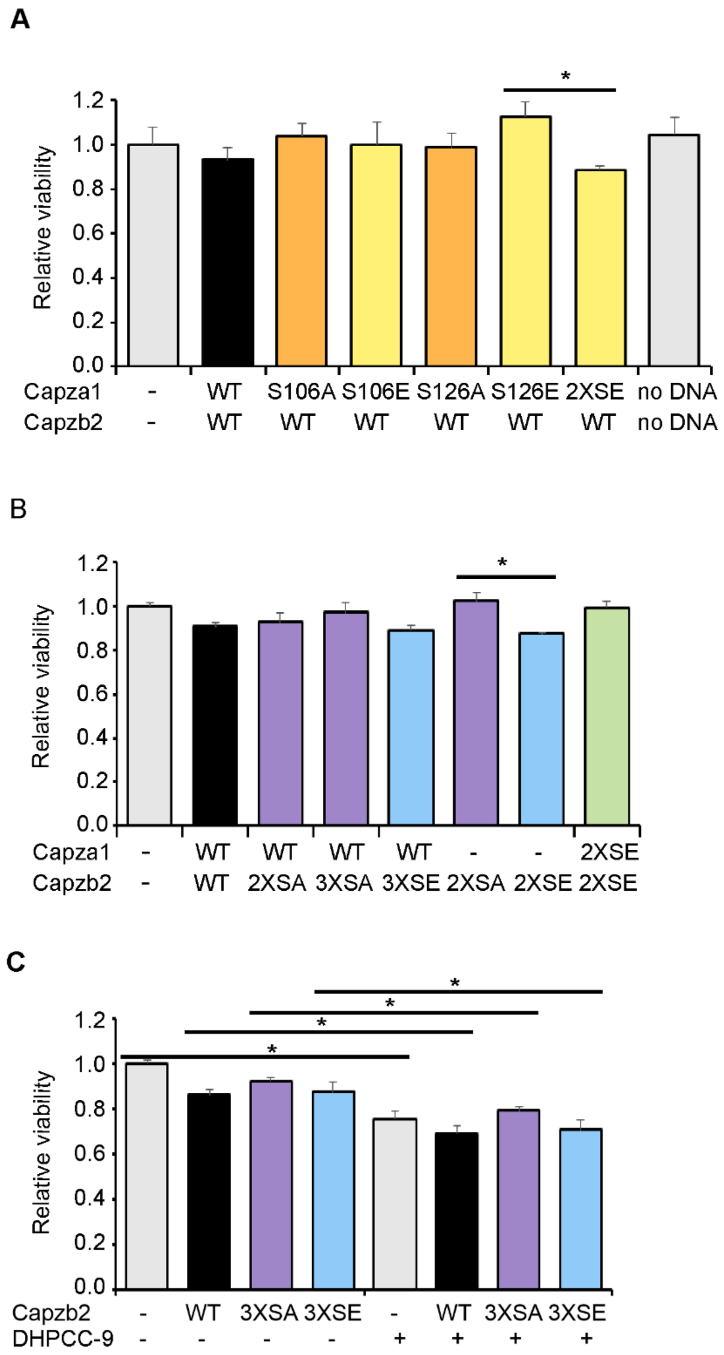

Figure S6 – CP phosphorylation does not have major effects on cell survival

(A-C) Wild-type (WT) Capza1 and Capzb2 or single phosphomimicking (SE) or phosphodeficient (SA) mutants or double (2X) or triple (3X) phosphomutants were transiently overexpressed in PC-3 cells. Cell motility was analysed by wound healing assays, which were initiated 12 h after transfection, and followed up for another 12 h, as shown in Figure 3. Cell viability was then measured by MTT assays. Shown are average viabilities from at least three independent assays.

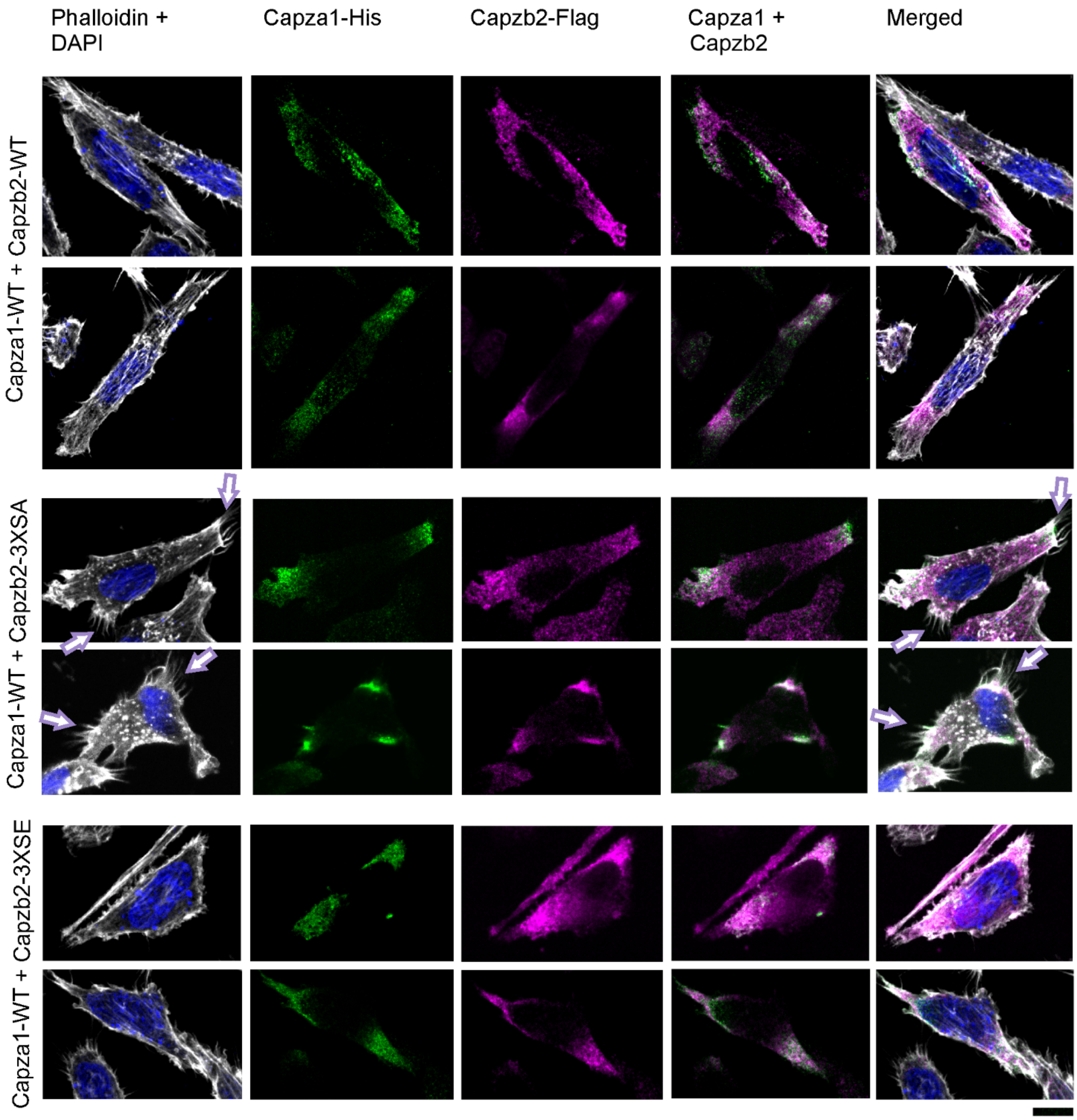

Figure S7 – CP dephosphorylation increases the number of actin protrusions

(A-C) PC-3 cells were transiently transfected to overexpress wild-type (WT) Capza1 and Capzb2 or their phosphodeficient (SA) or phosphomimicking (SE) mutants. Shown are images for the wild-type proteins as well as the phosphomutants. The increase in the number of actin protrusions in SA samples is pointed out by arrows. Scale bars represent 10 µm.

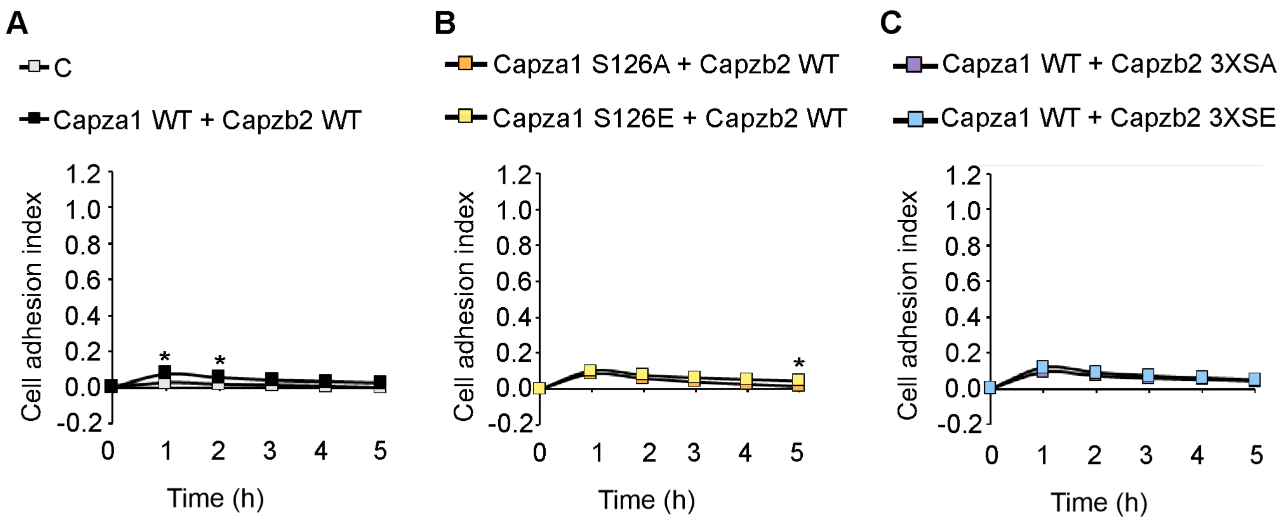

Figure S8 – Cell adhesion assay controls

(A-C) PC-3 cells were transiently transfected with wild-type (WT), phosphodeficient (SA) or phosphomimicking (SE) CPs. Cell adhesion to poly-L-lysine was measured according to electrical impedance. Three experiments were performed, while shown are results from one representative experiment with three parallel samples.

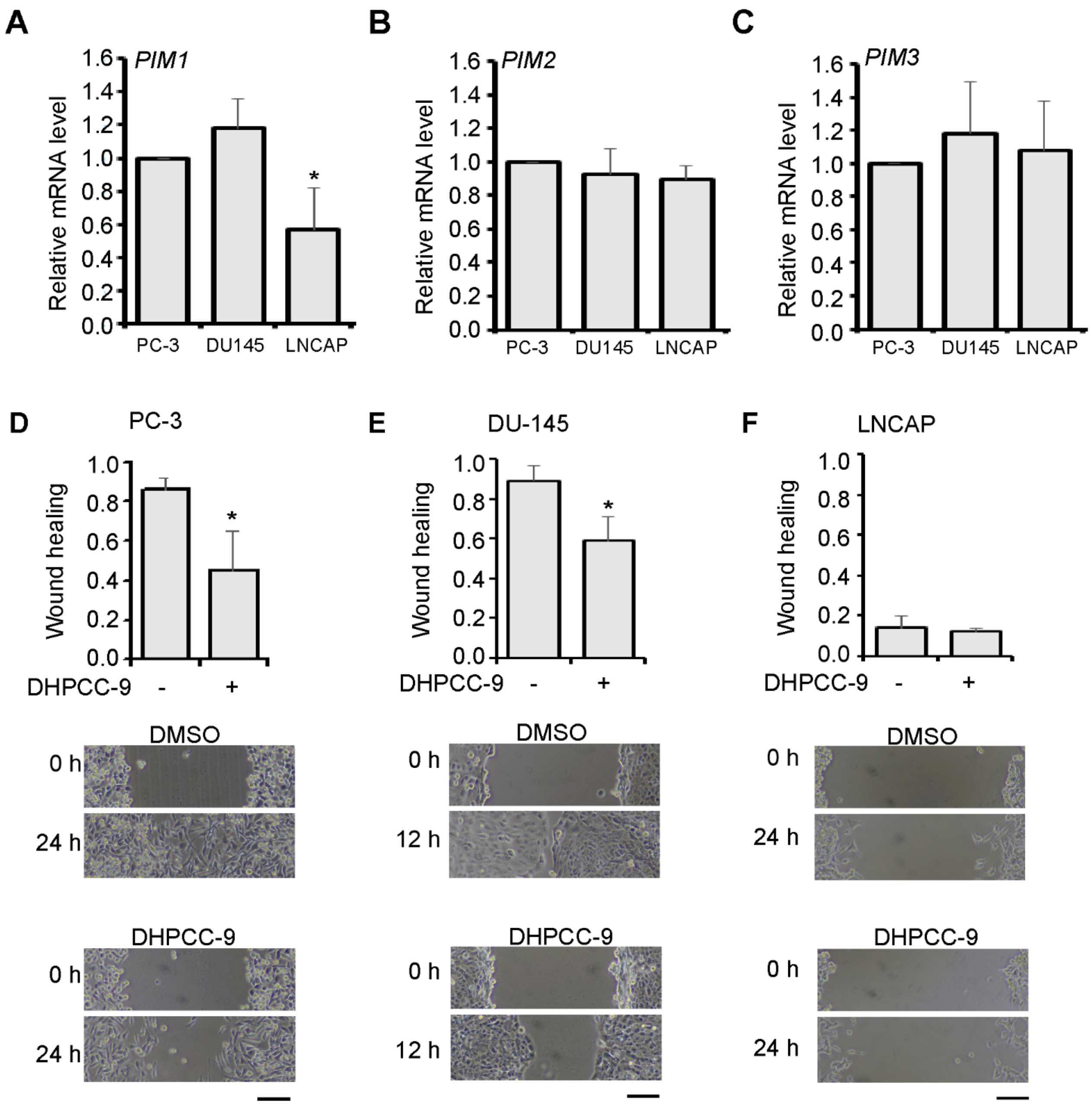

Figure S9 – Prostate cancer cell migration is reduced by PIM inhibition in a cell line-dependent manner

(A-C) *PIM* mRNA levels were measured in untransfected prostate cancer cell lines by RT-PCR and compared to those of PC-3 cells. **(D-F)** Wound healing assays were performed with the same cell lines treated with DMSO or 10 µM DHPCC-9. Wound closure was followed for 12 to 24 h, and shown are average results from at least three independent experiments with two or three parallel samples along with representative examples. Scale bars represent 20 µm.

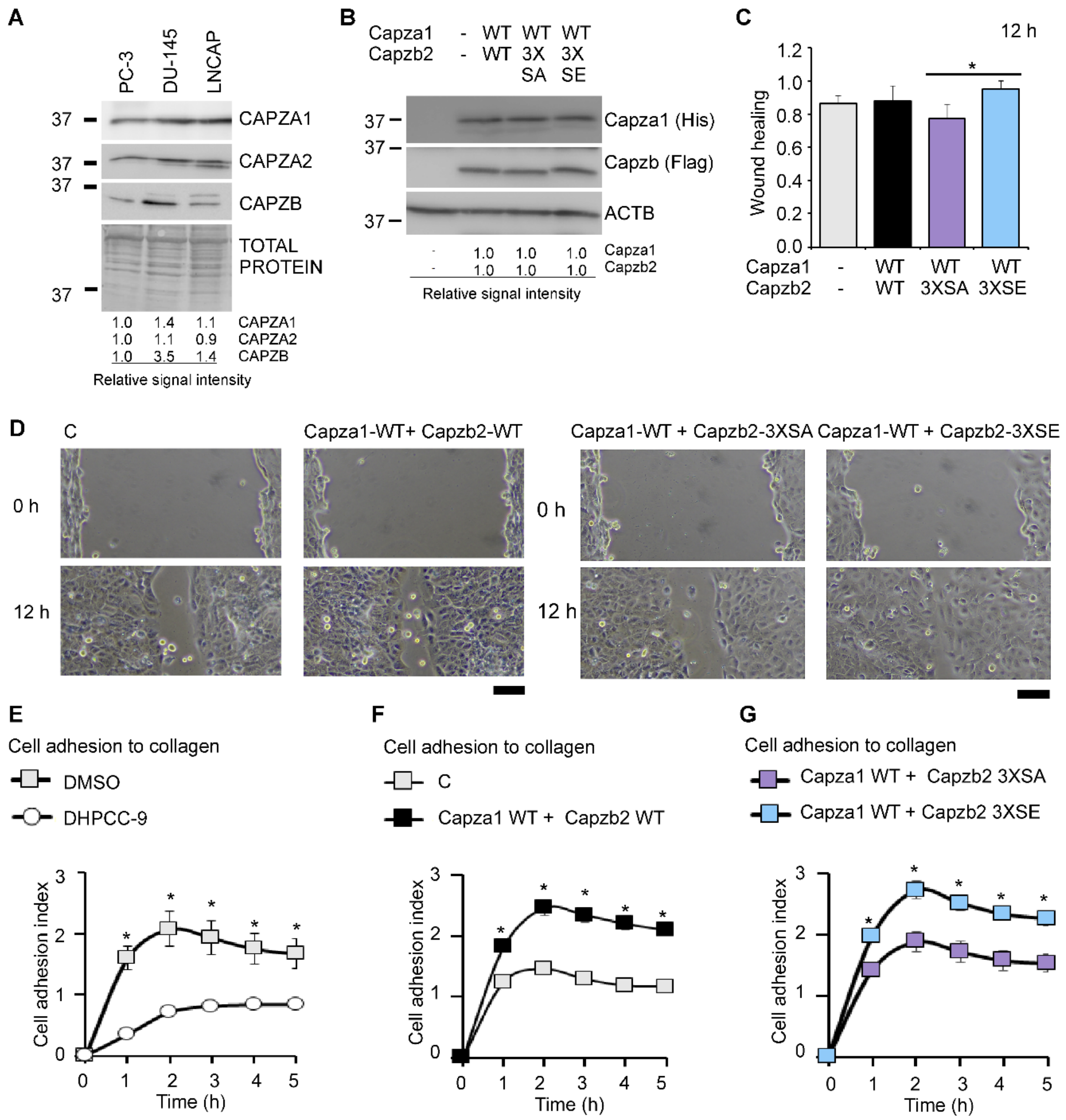

Figure S10 – CP phosphorylation promotes DU-145 cell migration and adhesion

(A-B) Western blotting was used to detect endogenous CPs in prostate cancer cell lines and transiently transfected CPs in DU-145 cells. **(C-D)** Cell migration was measured by wound healing assays from DU-145 cells overexpressing wild-type (WT) or phosphomutant (3XSA or 3XSE) CPs. Shown are average results from three independent experiments with representative images. Scale bars represent 20 μm. **(E)** Cell adhesion of untransfected or transiently transfected DU-145 cells was measured according to electrical impedance. PIM activity was inhibited by 10 μM DHPCC-9 starting 12 h prior to the assays. **(F-G)** The adhesion of transfected cells was measured starting 12 h after transfection. Similar experiments were performed at least three times with three parallel samples, while shown are representative results from one experiment.

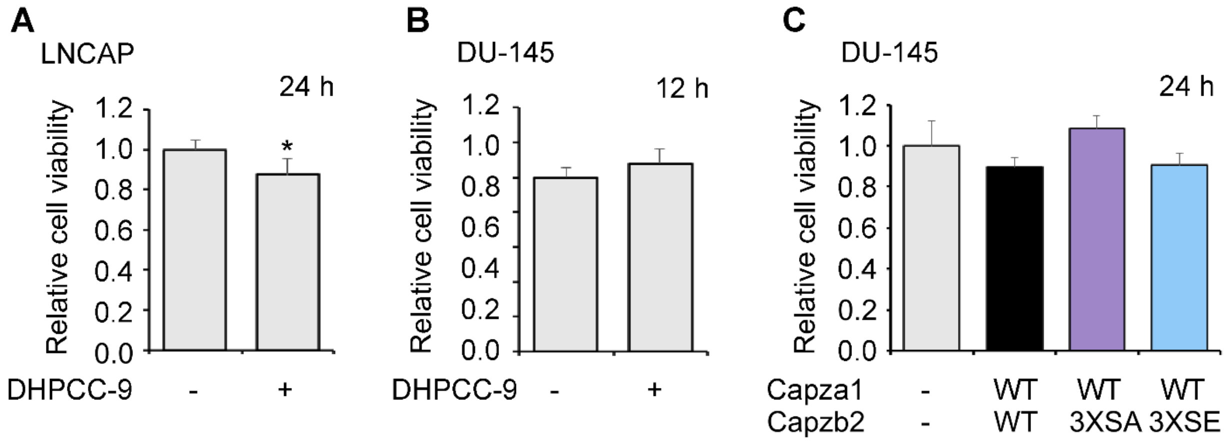

Figure S11 – Prostate cancer cell survival after different treatments or transfections

MTT assays were performed to the wound healing assay samples used for Figure S10. **(A-B)** Non-transfected LNCAP or DU-145 cells were measured 24 or 12 hours after the beginning of the treatment depending on the length of the wound healing assay**. (C)** Similarly, MTT assays were performed to transiently transfected DU-145 cells 24 hours after transfection.

A. Capza1 vs CAPZA1 sequence similarity

```
Mouse  1    MADFEDRVSDEEKVRIAAKFITHAPPGEFNEVFNDVRLLLNNDNLLREGAAHAFAQYNMD  60
            MADF+DRVSDEEKVRIAAKFITHAPPGEFNEVFNDVRLLLNNDNLLREGAAHAFAQYNMD
Human  1    MADFDDRVSDEEKVRIAAKFITHAPPGEFNEVFNDVRLLLNNDNLLREGAAHAFAQYNMD  60
                                                        S106
Mouse  61   QFTPVKIEGYDDQVLITEHGDLGNSRFLDPRNQISFKFDHLRKEASDPQPEDVDGGLKSW  120
            QFTPVKIEGY+DQVLITEHGDLGNSRFLDPRN+ISFKFDHLRKEASDPQPE+ DGGLKSW
Human  61   QFTPVKIEGYEDQVLITEHGDLGNSRFLDPRNKISFKFDHLRKEASDPQPEEADGGLKSW  120
                 S126
Mouse  121  RESCDSALRAYVKDHYSNGFCTVYAKTIDGQQTIIACIESHQFQPKNFWNGRWRSEWKFT  180
            RESCDSALRAYVKDHYSNGFCTVYAKTIDGQQTIIACIESHQFQPKNFWNGRWRSEWKFT
Human  121  RESCDSALRAYVKDHYSNGFCTVYAKTIDGQQTIIACIESHQFQPKNFWNGRWRSEWKFT  180

Mouse  181  ITPPSAQVVGVLKIQVHYYEDGNVQLVSHKDVQDSVTVSNEIQTTKEFIKIIESAENEYQ  240
            ITPP+AQVVGVLKIQVHYYEDGNVQLVSHKDVQDS+TVSNE QT KEFIKIIE+AENEYQ
Human  181  ITPPTAQVVGVLKIQVHYYEDGNVQLVSHKDVQDSLTVSNEAQTAKEFIKIIENAENEYQ  240

Mouse  241  TAISENYQTMSDTTFKALRRQLPVTRTKIDWNKILSYKIGKEMQNA  286
            TAISENYQTMSDTTFKALRRQLPVTRTKIDWNKILSYKIGKEMQNA
Human  241  TAISENYQTMSDTTFKALRRQLPVTRTKIDWNKILSYKIGKEMQNA  286
```

Uniprot identifier P47753-1 for mouse and P52907-1 for human

B. Capzb vs CAPZB sequence similarity

```
Mouse  1    MSDQQLDCALDLMRRLPPQQIEKNLSDLIDLVPSLCEDLLSSVDQPLKIARDKVVGKDYL  60
            MSDQQLDCALDLMRRLPPQQIEKNLSDLIDLVPSLCEDLLSSVDQPLKIARDKVVGKDYL
Human  1    MSDQQLDCALDLMRRLPPQQIEKNLSDLIDLVPSLCEDLLSSVDQPLKIARDKVVGKDYL  60

Mouse  61   LCDYNRDGDSYRSPWSNKYDPPLEDGAMPSARLRKLEVEANNAFDQYRDLYFEGGVSSVY  120
            LCDYNRDGDSYRSPWSNKYDPPLEDGAMPSARLRKLEVEANNAFDQYRDLYFEGGVSSVY
Human  61   LCDYNRDGDSYRSPWSNKYDPPLEDGAMPSARLRKLEVEANNAFDQYRDLYFEGGVSSVY  120

Mouse  121  LWDLDHGFAGVILIKKAGDGSKKIKGCWDSIHVVEVQEKSSGRTAHYKLTSTVMLWLQTN  180
            LWDLDHGFAGVILIKKAGDGSKKIKGCWDSIHVVEVQEKSSGRTAHYKLTSTVMLWLQTN
Human  121  LWDLDHGFAGVILIKKAGDGSKKIKGCWDSIHVVEVQEKSSGRTAHYKLTSTVMLWLQTN  180
            S182       S192                                 S226
Mouse  181  KSGSGTMNLGGSLTRQMEKDETVSDCSPHIANIGRLVEDMENKIRSTLNEIYFGKTKDIV  240
            KSGSGTMNLGGSLTRQMEKDETVSDCSPHIANIGRLVEDMENKIRSTLNEIYFGKTKDIV
Human  181  KSGSGTMNLGGSLTRQMEKDETVSDCSPHIANIGRLVEDMENKIRSTLNEIYFGKTKDIV  240

Mouse  241  NGLRSVQTFADKSKQEALKNDLVEALKRKQQC  272
            NGLRSVQTFADKSKQEALKNDLVEALKRKQQC
Human  241  NGLRSVQTFADKSKQEALKNDLVEALKRKQQC  272
```

Uniprot identifier P47757-2 for mouse and P47756-2 for human

Figure S12 – Comparison of human and mouse CP sequences

(A-B) BLAST was used to compare sequence similarities between mouse and human CP subunits at the PIM1 phosphorylation target sites (in bold and marked above the sequences) and their surrounding areas (inside squares).
